# Umbelliferone and Its Synthetic Derivatives as Suitable Molecules for the Development of Agents with Biological Activities: A Review of Their Pharmacological and Therapeutic Potential

**DOI:** 10.3390/ph16121732

**Published:** 2023-12-15

**Authors:** Anita Kornicka, Łukasz Balewski, Monika Lahutta, Jakub Kokoszka

**Affiliations:** Department of Chemical Technology of Drugs, Faculty of Pharmacy, Medical University of Gdansk, 80-416 Gdansk, Poland; lukasz.balewski@gumed.edu.pl (Ł.B.); monika.lahutta@gumed.edu.pl (M.L.); jakub.kokoszka@gumed.edu.pl (J.K.)

**Keywords:** umbelliferone, 7-hydroxycoumarin-based compounds, pharmacological properties, fluorescence probes

## Abstract

Umbelliferone (UMB), known as 7-hydroxycoumarin, hydrangine, or skimmetine, is a naturally occurring coumarin in the plant kingdom, mainly from the *Umbelliferae* family that possesses a wide variety of pharmacological properties. In addition, the use of nanoparticles containing umbelliferone may improve anti-inflammatory or anticancer therapy. Also, its derivatives are endowed with great potential for therapeutic applications due to their broad spectrum of biological activities such as anti-inflammatory, antioxidant, neuroprotective, antipsychotic, antiepileptic, antidiabetic, antimicrobial, antiviral, and antiproliferative effects. Moreover, 7-hydroxycoumarin ligands have been implemented to develop 7-hydroxycoumarin-based metal complexes with improved pharmacological activity. Besides therapeutic applications, umbelliferone analogues have been designed as fluorescent probes for the detection of biologically important species, such as enzymes, lysosomes, and endosomes, or for monitoring cell processes and protein functions as well various diseases caused by an excess of hydrogen peroxide. Furthermore, 7-hydroxy-based chemosensors may serve as a highly selective tool for Al^3+^ and Hg^2+^ detection in biological systems. This review is devoted to a summary of the research on umbelliferone and its synthetic derivatives in terms of biological and pharmaceutical properties, especially those reported in the literature during the period of 2017–2023. Future potential applications of umbelliferone and its synthetic derivatives are presented.

## 1. Introduction

Phytochemicals constitute a large group of bioactive compounds derived from natural resources, especially those of plant origin. Among them, coumarins containing a 2*H*-1-benzopyran-2-one core found in a wide range of plants demonstrate the broad spectrum of pharmacological properties, including anticancer, antimicrobial, antiviral, anticoagulant, antihypertensive, anti-inflammatory, and antioxidant or neuroprotective activities [1]. 

**Figure 1:**
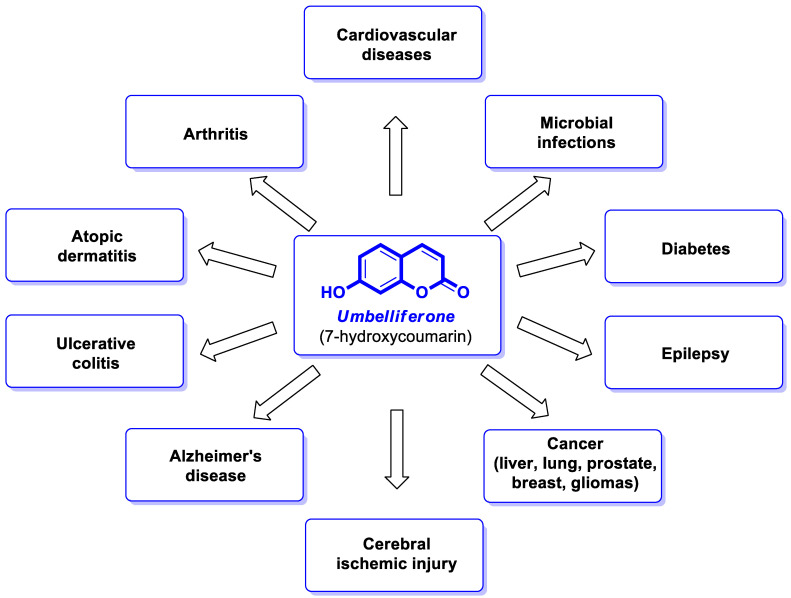
Therapeutical potential of umbelliferone [3,4,5].

Umbelliferone (UMB) (Figure 1), also known as 7-hydroxycoumarin, hydrangine, or skimmetine, is one of the most common plant-based coumarins present as a secondary metabolite in the flowers, fruits, and roots of almost all higher plants, mainly from the *Umbelliferae*/*Apiaceae* family [2]. The potential therapeutic effects of UMB in diabetes, cardiovascular or neurodegenerative diseases, inflammatory disorders, various cancer types, and microbial infections [3,4,5] (Figure 1) have gained increasing interest in the development of its synthetic derivatives with beneficial pharmacological activities. 

**Figure 2:**
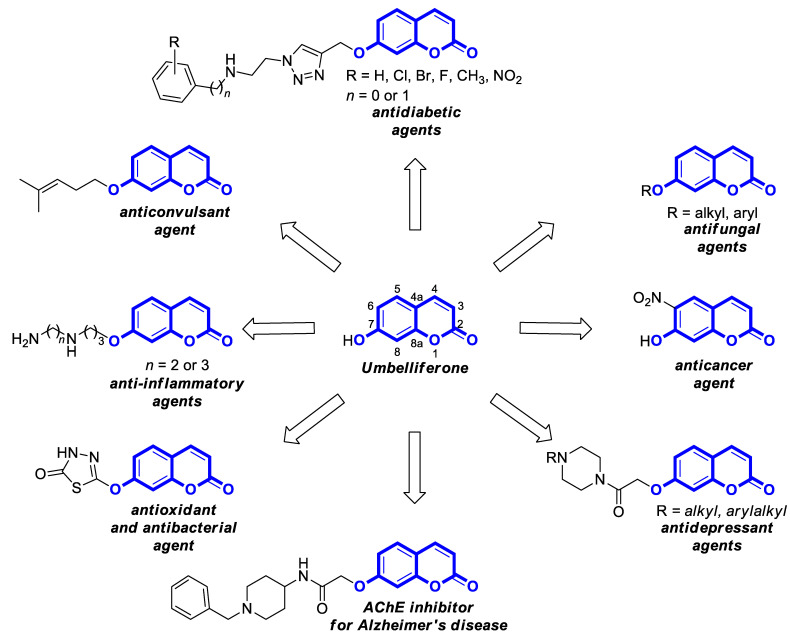
Biologically active 7-hydroxycoumarins derived from umbelliferone [3,6,7,8,9,10,11].

In addition, an accessible scaffold for transformation into various biologically active functionalized 7-hydroxycoumarins (Figure 2) [3,4,6,7,8,9,10,11] along with the lack of oral toxicity within the dose range of 200 mg/kg [12,13,14] make umbelliferone an attractive platform for the development of bioactive 7-hydroxycoumarin-based compounds in drug design.

This review aimed to highlight the recent advances in the development of newly synthesized 7-hydroxycoumarin-based compounds including metal complexes with anti-inflammatory, antioxidant, antineurodegenerative, antipsychotic, antiepileptic, antidiabetic, and chemotherapeutic activities as well as fluorescence properties especially over the past seven years. Because the pharmacological properties of umbelliferone have been extensively reviewed previously [3,4,5], the drastically selected studies will be discussed here in relation to umbelliferone. 

Research articles and reviews used for the preparation of this manuscript were collected by using several electronic databases, including SciFinder, PubMed, Web of Science, and Scopus.

## 2. Anti-Inflammatory Activity

### 2.1. Anti-Inflammatory Properties of Umbelliferone

Inflammation is part of a complex biological process in the human body caused by various stimuli including pathogenic microorganisms, cell damage, irritants, or immune reactions. Because this process is necessary to protect the body, it should lead to the removal of pathogens and allow the tissue to return to its physiological state. On the other hand, prolonged inflammation is associated with the development of minor-to-major diseases such as rheumatoid arthritis, chronic asthma, multiple sclerosis, inflammatory bowel disease, or psoriasis, as well as cancer [15,16]. 

Similar to other natural coumarins including scopoletin, visnadin, marmin, daphnethin, or esculetin, umbelliferone also exhibits a favorable anti-inflammatory effect via various inflammatory signaling pathways [3,5,17,18,19]. 

In allergic conditions, the increase in NO production is associated with the severity of allergic symptoms, and its generation is regulated by inducible nitric oxidase synthase (iNOS) genes [20,21]. In turn, Nrf2 (nuclear factor erythroid 2 (NEF)-related factor 2) is a key signaling pathway involved in the regulation of the endogenous antioxidant system formed by heme oxygenase-1 (HO-1), superoxide dismutase (SOD), catalase (CAT), nicotinamide adenine dinucleotide phosphate (NADPH) oxidase (NOX), as well as thioredoxin and it protects cells from the oxidative stress markers [22]. Moreover, Nrf2 can reduce the inflammatory process by the inhibition of the production and the release of pro-inflammatory cytokines [23].

It has been reported that intraperitoneal administration of 1, 10, and 50 mg/kg of umbelliferone in BALB/c mice significantly attenuated both acute histamine- and chronic picryl chloride-induced ear edema reducing the allergic symptoms and the oxidative stress by the induction of the Nrf2 expression on the one hand and downregulation of iNO expression on the other hand [24].

The therapeutic potential of umbelliferone on ulcerative colitis response and an oxidative injury induced via the intrarectal administration of acetic acid in rats was evaluated [25]. The efficacy of umbelliferone in alleviating ulcerative colitis was associated with downregulation of the TLK4/NF-κB-p65/iNOS signaling pathway, which led to a reduction in the expression of the pro-inflammatory cytokines such as TNF-α, IL-6, and MPO. Furthermore, umbelliferone protected rats against acetic acid-induced ulcerative colitis through upregulation of SIRT1/PPARγ signaling with subsequent inhibition of NF- NF-κB-p65 activity or downregulation of the p38MAPK/ERK signaling in addition to preventing reactive oxygen species (ROS) generation [25].

Umbelliferone sourced from *Saussurea laniceps* along with scopoletin has been identified as a major anti-rheumatic component of this herb that combats rheumatoid arthritis (RA) [26]. It was demonstrated that umbelliferone exhibits anti-rheumatoid activity via a multitarget mechanism of action. Thus, it can bind and inhibit tyrosine kinases on fibroblast-like synoviocytes, the pivotal effector cells in RA, to block their proliferation, migration, and invasion. On the other hand, targeting tyrosine kinases leads to the blockage of NF-κB signaling which mediates the inflammatory signaling cascade. Therefore, the umbelliferone scaffold could be used to develop multitarget anti-rheumatoid drugs [26]. Moreover, umbelliferone attenuated the severity of collagen-induced arthritis in rats by inhibiting proliferation and inducing apoptosis of fibroblast-like synoviocytes as a result of the downregulation of the Wnt/β-catenin signaling pathway [27]. 

Notably, gelatin-coated ZnO-ZnS core-shell nanoparticles with umbelliferone improved arthritis therapy when intravenously injected into collagen-induced arthritis rats by reducing the production of pro-inflammatory cytokines such as interleukin-1β (IL-1β), IL-6, and IL-17, as well as prostaglandin E (PEG2) [28].

Umbelliferone was also reported as a potential therapeutic agent of atopic dermatitis (AD). In 2019, Ji-ye et al. demonstrated that oral administration of umbelliferone reduces 2,4-dinitrochlorobenzene (DNCB)/*Dermatophagoides farinae* extract (DFE)-induced atopic dermatosis symptoms in mice by suppressing pro-inflammatory cytokines and chemokines [29]. In the same study, 7-hydroxycoumarin was shown to suppress the secretion of pro-inflammatory cytokines and chemokines in TNF-α/IFN-γ-treated HaCAT cells by inhibiting IκBα degradation, the nuclear translocation of NF-κB, and the phosphorylation of STAT1 in a dose-dependent manner [29].

Noteworthy is the increase in the transdermal permeation and anti-inflammatory potential of umbelliferone by employing an umbelliferone–phospholipids complex-loaded matrix film (UPLC-MF) [30]. The anti-inflammatory efficacy of umbelliferone and improved physicochemical properties of the combined formulation system resulted in the significant enhancement of edema inhibition in the carrageenan-induced Albino rat paw model by restoring or minimizing the infiltration of inflammatory cells such as neutrophils and mononuclear cells to normal cells. The obtained results suggested that the prepared formulation system has potential as a promising strategy for improving the transdermal penetration of 7-hydroxycoumarin [30].

### 2.2. Synthetic 7-Hydroxycoumarin-Based Compounds as Anti-Inflammatory Agents

Given its favorable anti-inflammatory activity, the umbelliferone framework has been used for chemical modification to identify original and effective compounds that can serve as anti-inflammatory agents [17,31]. 

**Figure 3:**
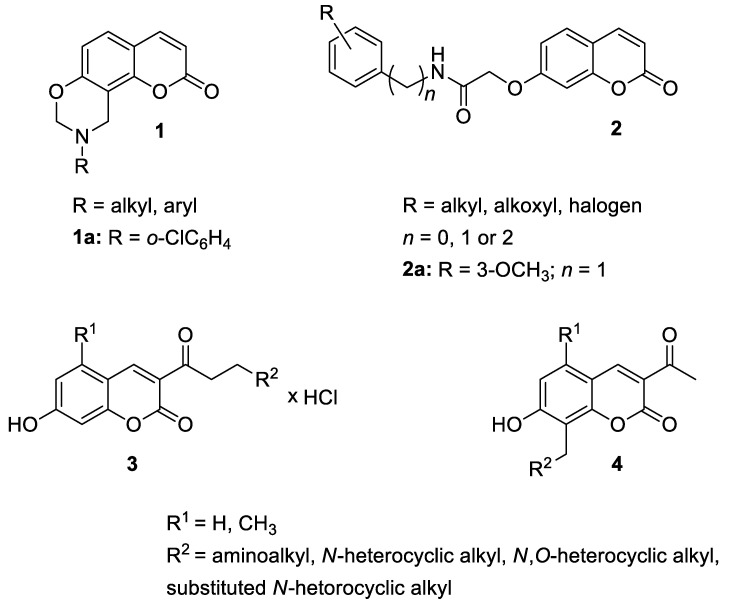
Anti-inflammatory 7-hydroxycoumarin-based compounds **1**–**4**.

Recently, 9,10-dihydrochromeno[8.7-*e*][1,3]oxazin-2(8*H*)-one derivatives (**1**) were designed and synthesized as potential anti-inflammatory agents (Figure 3) [32].

**Figure 4:**
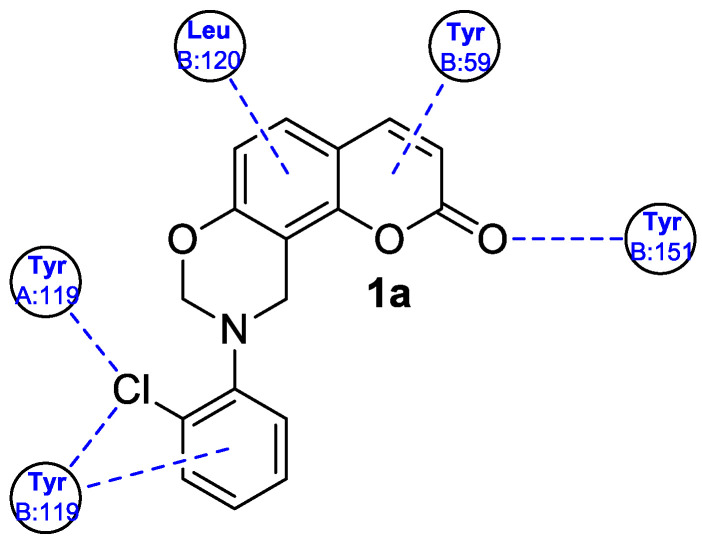
A 2D representation of docked ligand **1a** in TNF-α.

Among the compounds that showed anti-inflammatory activity, analogue **1a** has been found to exert the most potent biological effect, which was determined to be capable of decreasing the concentration of pro-inflammatory cytokines including TNF-α and IL-6 in lipopolysaccharide (LPS)-induced cytokine release in RAW264.7 mouse macrophages. It has been indicated that derivative **1a** can inhibit inflammatory responses by suppressing the MAPK (mitogen-activated protein kinase) and NK-*κ*B signaling pathways that play a pivotal role in the regulation of inflammatory cytokines [33]. Moreover, the results of the docking study for compound **1a** within TNF-α showed the H-bond formation between the carbonyl group of the coumarin ring and the side chain of Tyr151 and π-π interactions between the benzene ring of derivative **1a** and the side chain of Tyr119, possibly enhancing its inhibitory activity (Figure 4) [32].

**Figure 5:**
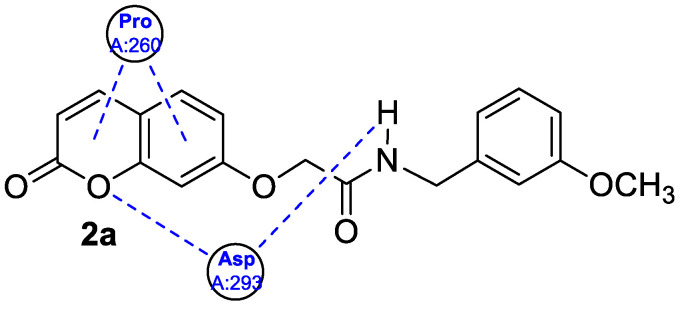
A 2D model of the interaction between 7-hydroxycoumarin derivative **2a** with the active site of NF-κB p65.

The newly synthesized 2-[(2-oxo-2*H*-chromen-7-yl)oxy]acetamides of general formula **2** (Figure 3) hybridized with substituted aniline or benzylamine moieties were also explored for their potential anti-inflammatory activity against LPS-induced IL-6 and TNF-α release in RAW264.7 cells [34]. Among the tested compounds, *N*-(3-methoxybenzyl)-2-[(2-oxo-2*H*-chromen-7-yl)oxy]acetamide (**2a**) (Figure 3) proved to be the most active. It was found that compound **2a** can exert its anti-inflammatory activity by reducing the levels of IL-6 and TNF-α via blocking the NF-*κ*B signaling pathways. The docking study showed the appropriate interactions between coumarin **2a** and NF-*κ*B. It was revealed that compound **2a** can bind to the active site (NLS Peptide) of NF-*κ*B p65, which was confirmed by surface plasmon resonance (SPR) analysis (Figure 5). The analogue **2a** was found to have high binding affinity to NF-*κ*B p65 with a KD value of 2.83 *×* 10^−7^ M [34].

Additionally, in 2021, Gao et al. used the Knoevenagel reaction and Pechmann condensation to develop a new series of 3-acetyl-7-hydroxycoumarin Mannich bases (**3**) and Betti bases (**4**) (Figure 3) that were explored in vitro for their anti-inflammatory activity [35]. The obtained results showed that the Mannich bases (**3**) possess much stronger anti-inflammatory properties than the corresponding Betti bases (**4**) in both the NO release and TNF-α production models. Moreover, the structure–activity relationship analysis (SAR) revealed that the presence of the diethylamino group is beneficial to the anti-inflammatory activity [35].

## 3. Antioxidant Activity

### 3.1. Antioxidant Properties of Umbelliferone

Oxidative stress is implicated in a number of pathological conditions such as cardiovascular diseases, cancer, neurodegenerative diseases, diabetes mellitus, ischemia/reperfusion injury, or rheumatoid arthritis, as well as in the ageing process through multiple mechanisms, where free radicals contribute to cellular damage [36]. Therefore, there is a growing interest in antioxidant agents with therapeutic potential [37].

In this line, the antioxidant potential of umbelliferone is also worth mentioning. As was nicely elaborated by Mazimba [3] and Lin et al. [5], its antioxidant properties are associated with the ability to scavenge free radicals as well as the inhibition of lipid peroxidation. Moreover, as mentioned in Section 2.1, the antioxidant effect of umbelliferone can be a result of the activation of the Nrf2 signaling pathway leading to the increasing activities of endogenous antioxidant substances such as superoxide dismutase (SOD), glutathione (GSH), catalase (CAT), and NAD(P)H-quinone oxidoreductase (NQO1) [5,22].

Recently, umbelliferone was also found to inhibit ferroptosis, a novel form of lipid reactive oxygen species and iron-dependent cell death. It has been claimed that umbelliferone may delay the progression of diabetic nephropathy, possibly by activating the Nrf2/heme-oxygenase-1 pathway, thereby reducing the level of high glucose-induced ferroptosis [38].

### 3.2. Synthetic 7-Hydroxycoumarin-Based Compounds as Antioxidant Agents

**Figure 6:**
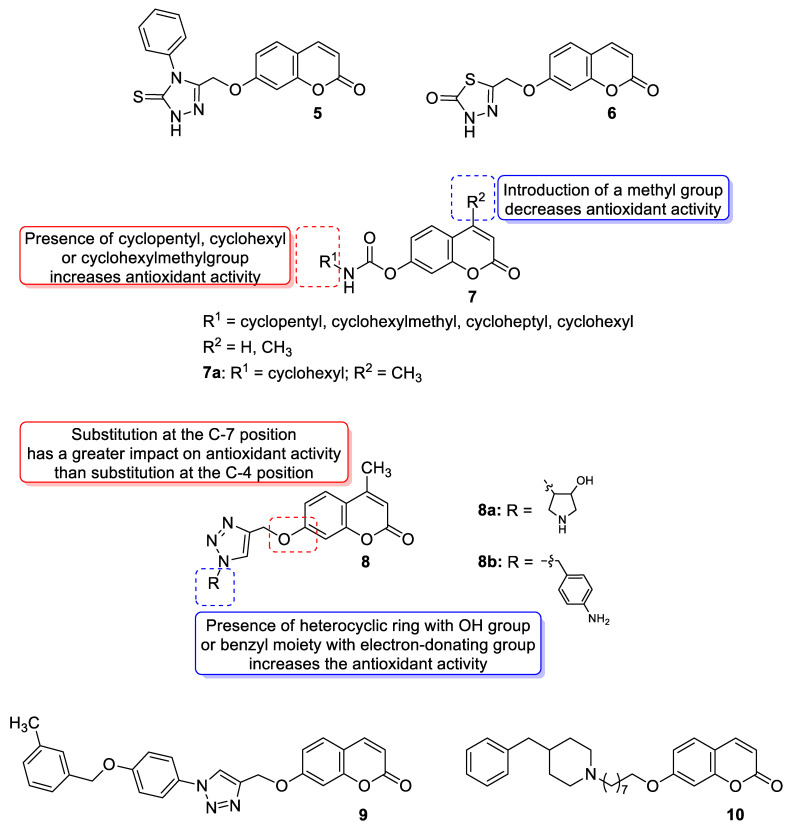
Antioxidant 7-hydroxycoumarin-based compounds **5**–**10**.

With regards to the antioxidant activity of 7-hydroxycoumarin-based compounds, Al-Majedy et al. designed and synthesized two series of modified 7-hydroxycoumarins and evaluated them for their antioxidant potency [39,40]. Among them, the best radical scavenging properties were shown by 7-[(4-phenyl-5-thioxo-4,5-dihydro-1*H*-1,2,4-triazol-3-yl)methoxy]coumarin (**5**) and 5-{[(coumarin-7-yl)oxy]methyl}-1,3,4-thiadiazol-2(3*H*)-one (**6**) (Figure 6), which exhibited the inhibition of 91% and 88% of free radicals, respectively, at a concentration of 250 µg/mL in the 2,2′-diphenyl-1-picrylhydrazyl radical assay (DPPH) [40].

In 2018, Kurt et al. evaluated novel coumarin carbamate derivatives (**7**) (Figure 6) for their anticholinesterase, antioxidant, and anti-aflatoxigenic activities [41]. The synthesized compounds exhibited moderate-to-low radical scavenging ability (IC_50_ = 23.15–>200 µM) in 2,2′-azino-bis-3-ethylbenzothiazoline-6-sulfonic acid (ABTS) analysis compared to *quercetin* (IC_50_ = 15.49 µM) used as a standard compound. The SAR analysis showed that the presence of cyclopentyl, cyclohexyl, or cyclohexylmethyl at the R^1^ position of the carbamate moiety increased antioxidant potential compared to the cycloheptyl group (IC_50_ = 66.80–80.03 µM vs. IC_50_ = 131.85 µM). Furthermore, a two-fold decrease in antioxidant potency was observed after the introduction of a methyl group at the R^2^ position of the coumarin scaffold. The exception to this was compound **7a** (R^1^ = cyclohexyl, R^2^ = CH_3_), which evidenced higher activity than other members of the tested derivatives (IC_50_ = 23.15 µM) (Figure 3) [41].

Regarding the antioxidant activity of 7-hydroxycoumarin-based compounds, some studies have been recently carried out showing the potential of coumarins linked with 1,2,3-triazoles [42,43]. Worth noting are coumarins of general formula **8** prepared by Joy et al. through the copper catalyzed azide-alkylene cycloaddition reaction (Figure 6) [42]. At a concentration of 100 μg/mL, compounds **8a** and **8b** exhibited comparable free radical inhibition to the standard drug—2,6-di-*tert*-butyl-4-methylphenol (BHT)—in the DPPH assay (74.2% and 73.5% vs. 88.6%). It was suggested that the promising antioxidant activity of these derivatives may be attributed to the presence of a herocyclic ring containing an OH group (**8a**: R = 4-hydroxypyrrolidin-3-yl) or a benzyl moiety bearing a NH_2_ substituent (**8b**: R = 4-aminobenzyl) [42].

Moreover, Kaushik and Chacal synthesized two series of coumarin-1,2,3-triazole hybrid molecules using the click chemistry approach from the coumarin-based terminal alkynes and aromatic azides and tested their antioxidant activity via the DPPH method [43]. However, all compounds displayed lower DPPH-based radical scavenging activity (IC_50_ = 3.33–8.75 μg/mL) compared to the standard ascorbic acid (IC_50_ = 1.23 μg/mL), and the presence of the electron-donating groups on the benzyl moiety in the structure of these compounds might contribute to increased antioxidant activity. In addition, the 7-hydroxycoumarin-based compounds generally evidenced higher activity than their 4-hydroxycoumarin-based counterparts. The best result was found for derivative **9** with an IC_50_ value of 3.33 μg/mL (Figure 6) [43].

Most recently, a new 7-hydroxycoumarin derivative **10** (Figure 6) was invented as a potential antioxidant agent [44]. Although the antioxidant activity of **10** was lower than the standard BHT (*p* < 0.05), it was found that all used concentrations (0.03125–1 mg/mL) owed its ability to scavenge radicals in the DPPH assay. The experimental antioxidant properties of coumarin **10** were also supported by molecular docking analysis that revealed the possible interactions of derivative **10** with the active binding site of CYP450. In addition, the DNA interaction studies showed that coumarin hybrid **10** can also act as an intercalator suggesting that this compound may be considered as a potential anticancer agent [44].

### 3.3. Metal Complexes with 7-Hydroxycoumarin-Based Compounds as Antioxidant Agents

As claimed in the earlier described review works, the 7-hydroxycoumarin-based metal complexes have a special ability to scavenge reactive oxygen species in biological systems [45,46].

**Figure 7:**
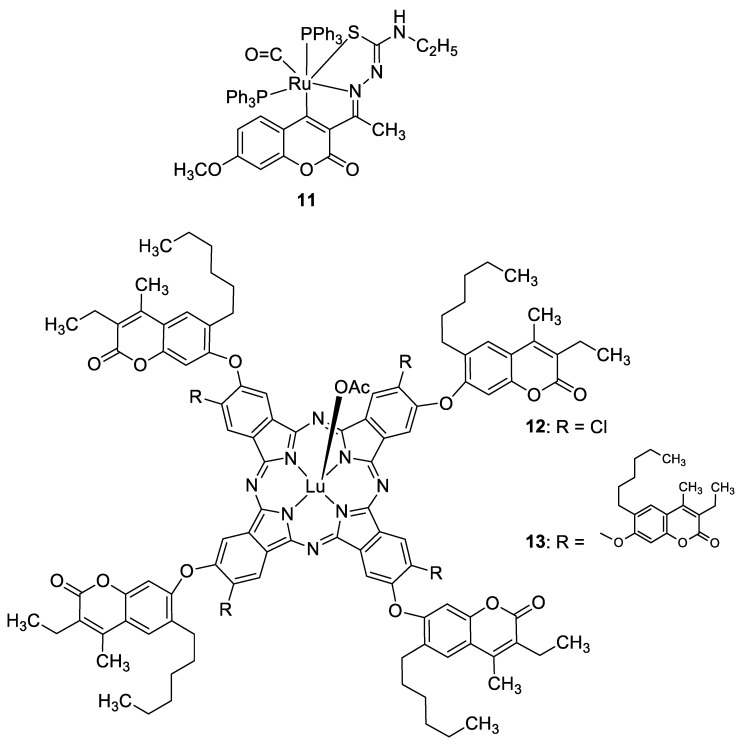
Antioxidant 7-hydroxycoumarin-based metal complexes **11**–**13**.

Recently, it was demonstrated that the radical scavenging ability of novel 3-acetyl-7-methoxy-4*N*-substituted thiosemicarbazones may be increased by ruthenium chelation [47]. The best radical scavenging properties have been shown by Ru(II) complex **11** (Figure 7), which displayed an antioxidant potency with about a fifteen-fold lower IC_50_ value than standard vitamin C in the DPPH model (IC_50_ = 5.28 µM vs. IC_50_ = 98.72 µM). Moreover, the results of the in vitro cytotoxic activity study revealed that complex **11** is a potent chemotherapeutic drug among the tested compounds. It was concluded that the promising antiproliferative potency might be attributed to the greater electron-donating ability of the *N*-terminal ethyl group [47].

In 2020, Özdemir et al. synthesized a series of 7-oxy-3-ethyl-6-hexyl-4-methylcoumarin-substituted lutetium(III) phthalocyanine compounds, whose antioxidant properties were evaluated [48]. Complexes **12** and **13** (Figure 7) displayed much better 2,2′-azino-bis-3-ethylbenzthiazoline-6-sulphonic acid (ABTS)-based radical cation scavenging activity compared with standard butylated hydroxyanisole (BHA), 120.344 mM troloxy/mg and 188.733 mM troloxy/mg vs. 52.63 mM troloxy/mg. On the other hand, the FRAP (Ferric Reducing Antioxidant Power) and CUPRAC (Cupric Reducing Antioxidant Capacity) analyses evidenced their lesser potency compared to BHT and vitamin C used as standards [48].

## 4. Umbelliferone and 7-Hydroxycoumarin-Based Compounds Acting in the Central Nervous System (CNS)

### 4.1. Neurodegenerative Disorders

The concept of an association between synaptic levels of acetylcholine in the brain, accumulation of β-amyloid plaques, neurofibrillary tangles, and Alzheimer’s disease (AD) has gained acceptance. Thus, the most common therapeutic approach for AD is the administration of cholinesterase inhibitors (ChEIs), which increase acetylcholine. In addition to the AChE concept, butyrylcholinesterase (BuChE) also plays a critical role in maintaining cholinergic function and selective BuChE inhibition has been regarded as a therapeutic approach in AD [49]. On the other hand, blocking aspartic protease β-secretase 1 (BACE1) that is responsible for selective proteolysis of amyloid precursor protein (APP) may be beneficial in AD treatment [50,51].

**Figure 8:**
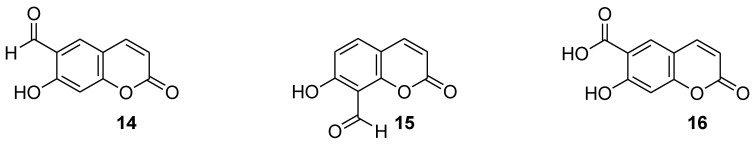
Umbelliferone derivatives **14**–**16** as potent AChE, BuChE, and BACE1 inhibitors for the treatment of neurodegenerative disorders.

Umbelliferone and its simple derivatives—6-formylumbelliferone (**14**) from the plant *Angelica decursiva*, its isomeric analogue 8-formylumbelliferone (**15**), and umbelliferone 6-carboxylic acid (**16**) (Figure 8)—exhibit potent inhibitory activities towards acetylcholinesterase (AChE), butyrylcholinesterase (BuChE), and aspartic protease β-secretase 1 (BACE1) [52,53,54]. However, it should be noted that the data regarding the inhibitory activity of umbelliferone towards AChE and BuChE are contradictory. In contrast to the aforementioned results, Lim et al. reported studies indicating an insignificant effect of umbelliferone on AChE and BuChE [5].

In turn, Hindam et al. supported that umbelliferone, by increasing glutathione content as well as reducing pro-inflammatory cytokines and acetylcholinesterase activity, exerts neuroprotective effects attenuating cognitive dysfunction in a *streptozotocin*-induced rat model of sporadic Alzheimer’s disease [55].

Recent studies carried out by Kurach et al. have also shown that umbelliferone improved cognitive dysfunction and enhanced memory by reducing the level of acetylcholinesterase in LPS-induced amnesia in a mice model [56].

**Figure 9:**
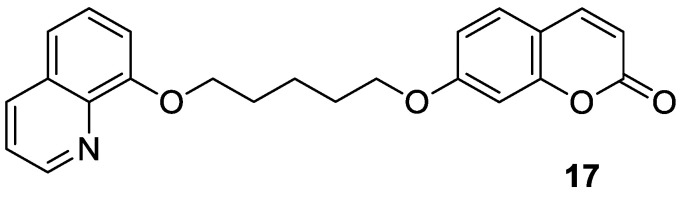
Chemical structure of 7-hydroxycoumarin-based compound **17** as an AChE and BuChE inhibitor.

Numerous diseases can be caused by a defect of more than one biological target—an enzyme or receptor. Thus, such disorders cannot be adequately addressed by the classical ‘one target, one molecule’ approach [57]. A promising strategy to tackle multifactorial diseases, e.g., AD, consists in the design of multifunctional agents, known as ‘hybrid’ molecules. These complex molecules display stable chemical combinations of two drug moieties or pharmacophores acting at different targets. Such ‘dual-acting compounds’ combine two distinct chemical entities [58,59]. According to this, Hirbod et al. designed a 7-hydroxycoumarin hybrid bearing a heterocyclic framework—8-hydroxyquinoline **17** (Figure 9)—as a novel cholinesterase inhibitor [60]. Conjugated with a five-membered CH_2_ chain, compound **17** showed pronounced AChE and BuChE inhibitory activity (IC_50_ = 8.8 µM and 26.5 µM, respectively). For the reference in this study, *donepezil*, the following IC_50_ values were achieved: 0.016 μM and 5.41 μM, respectively. The enzymatic assay for compound **17** was supported by docking studies at the AChE active site. Moreover, in silico optimization revealed that target ligand **17** interacts with the peripheral anionic site (PAS) of the enzyme, near the entry of the acetylcholinesterase gorge, and may prevent the formation of the stable AChE-amyloid-β peptide complexes. It has been claimed that conjugated 7-hydroxycoumarin derivative **17** is a promising cholinesterase inhibitor for further development [60].

It has been documented that dual inhibition of monoamine oxidase (MAO) and cholinesterase enzymes, which are complicit in AD’s complex pathophysiology, improve cognitive functions, achieve neuroprotectivity, and subsequently halt disease progression. Therefore, dual-functional cholinesterase and MAO inhibitors are emerging as a promising treatment option for AD [61,62].

**Figure 10:**
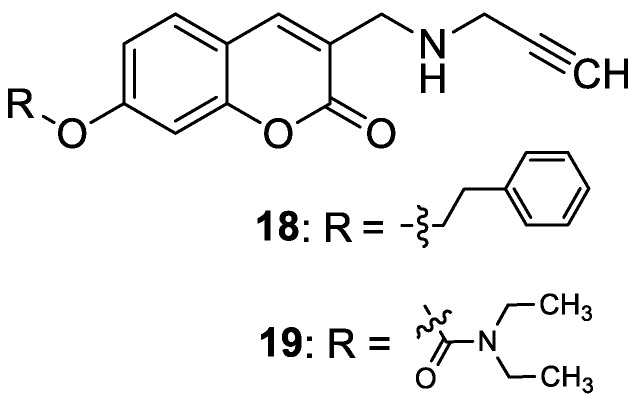
Chemical structures of 7-hydroxycoumarin-based compounds **18** and **19** as multifunctional anti-Alzheimer’s disease agents.

Recently, Mzezewa et al. have described 3-substituted 7-hydroxycoumarin derivatives **18** and **19** as multifunctional anti-Alzheimer’s disease agents (Figure 10) [63].

In addition, these compounds offer significant neuroprotective effects towards MPP^+^-compromised SH-SY5V neuroblastoma cells with no inherent cytotoxicity at 10 µM. Consequently, compounds **18** and **19** have been proposed for further studies to explore their neuroprotective potential in AD and related neurodegenerative diseases such as Parkinson’s disease. Although 7-hydroxycoumarins **18** and **19** exhibited weak cholinesterase inhibitory activity when compared with the reference *denezepril* (AChE and BuChE IC_50_ = >100 µM vs. AChE IC_50_ = 0.007 µM and BuChE IC_50_ = 4.40 µM), the tested compounds demonstrated selectivity towards MAO-B with IC_50_ values of 0.029 µM and 0.101 µM, respectively. The MAO-B selectivity index ratio, estimated as IC_50_ (MAO-A)/IC_50_ (MAO-B), for compound **18** is 133.2, and for derivative **19**, it equals 205.9. The structure–activity relationship analysis confirmed that the inhibitory capacity of 7-hydroxycoumarin derivatives **18** and **19** towards MAO-B is attributed to the substitution at the C-7 position of the coumarin scaffold. Moreover, addition of the propargylamine group (-NH-CH_2_-C≡CH) at the C-3 position of the designed compounds confers superior inhibition activity and selectivity [63].

Notably, compounds previously described (**7**, Figure 6) bearing a cyclohexylmethyl group (R^1^ = cyclohexylmethyl, R^2^ = H or CH_3_), in addition to their antioxidant properties, also displayed a strong inhibitory effect against butyrylcholinesterase with IC_50_ values of 0.003 µM and 0.004 µM. These IC_50_ values are almost 35,000-fold more than that of parent 7-hydroxycoumarin (IC_50_ = 105 µM), 5416-fold more than that of *galantamine* (IC_50_ = 16.25 µM), and 340-fold more potent than that of *rivastigmine* used as reference compounds [41].

### 4.2. Neuropsychiatric Diseases

#### 4.2.1. Synthetic 7-Hydroxycoumarin-Based Compounds Targeting Monoamine Oxidase (MAO) and D-Amino Acid Oxidase (DAAO)

There is increasing evidence that the free radical-mediated CNS neuronal dysfunctions are related not only to the pathophysiology of neurodegenerative but also neuropsychiatric disorders such as schizophrenia [64]. In this context, it must be stated that the activation of monoamine oxidase isoenzymes MAO-A and MAO-B catalyzes the α-carbon oxidation of amines followed by the formation of hydrogen peroxide and aldehydes as by-products. Hydrogen peroxide is involved in oxidative damage and apoptotic pathways leading to the necrosis and apoptosis of cells, and elevated levels of hydrogen peroxide and other reactive oxygen species (ROS) were found in aging organs such as the brain or heart. In addition, aldehyde derivative by-products cause synuclein aggregation, playing a crucial role in Parkinson’s disease. Hence, these data clearly suggest that the inhibition of monoamine oxidase MAO-A and MAO-B may be beneficial in the treatment of neurodegenerative and neuropsychiatric disorders, and compounds containing the benzopyran-2-one scaffold have been proposed as potent monoamine inhibitors (MAOIs) [64].

Recently, Seong et al. reported 6-formylumbelliferone derivative **14** and its isomeric analogue **15**, presented in Figure 8, as highly selective *h*MAO-A inhibitors [65]. The higher selectivity and inhibitory activity towards *h*MAO-A exhibited 7-hydroxy-2-oxo-2*H*-chromene-6-carbaldehyde (**14**) with an IC_50_ value of 3.23 μM for *h*MAO-A and an IC_50_ value of 15.31 μM for *h*MAO-B. Enzyme kinetic studies revealed that both 6-formylumbelliferone **14** and 8-formylumbelliferone **15** are competitive *h*MAO inhibitors. These investigations were supported by molecular docking studies. Data revealed that compounds **14** and **15** dock well into the active sites of recombinant human monoamine oxidase A and B. The formyl group of **14** interacts strongly with substrate binding site (SBS) residues Tyr444 and Tyr197 of *h*MAO-A via water-mediated hydrogen bonds, whereas Phe352 and Tyr407 residues are involved in hydrophobic noncovalent π-π T-shaped (perpendicular T-shaped) and π-π stacking interactions. Hydroxycoumarin derivatives **14** and **15** demonstrated a neuroprotective effect due to their antilipid peroxidation and anti-Aβ_25–35_ (amyloid β self-assembly) aggregation activity in rat brain tissue. The selectivity index (SI) calculated as the ratio the of IC_50_ of *h*MAO-A and *h*MAO-B for compound **14** is 0.21, and for compound **15**, it is 0.13. Formylumbelliferones **14** and **15** are possible scaffolds for developing multitarget-directed ligands in the treatment of neuropsychiatric diseases due to their neuroprotective effect via the inhibition of *h*MAO-A/B, self-Aβ aggregation, and lipid peroxidation. However, there is a need for more studies to confirm the mechanism of action in animal models, which could provide new insights into the potential of these compounds in the treatment of neuropsychiatric diseases. Further research is also necessary to evaluate the pharmacological and toxicological profiles of these molecules [65].

**Figure 11:**
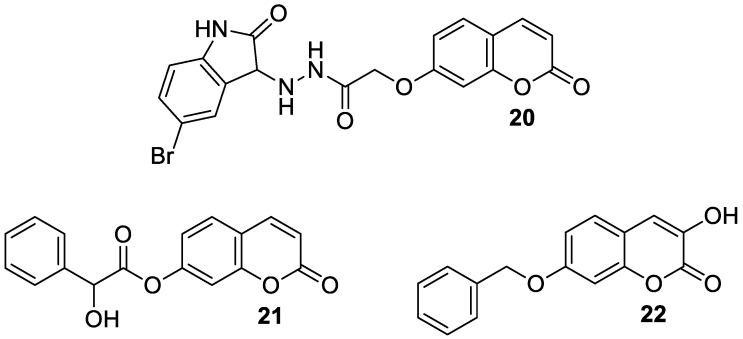
Chemical structures of 7-hydroxycoumarin-based compounds **20**–**22** as MAO and DAAO inhibitors.

In a study in 2018, Dhirman et al. investigated monoamine oxidase’s inhibitory effects on a series of umbelliferone-based compounds [66]. By substituting the coumarin scaffold at the C-7 position, MAO’s inhibitory potential was significantly increased. MAO inhibition studies have shown that hybrid compounds containing the 5-bromoisatin moiety **20** (Figure 11) exhibited a pronounced *h*MAO-A activity (IC_50_ = 7.47 μM), whereas incorporation of the 2-hydroxy-2-phenylacetate moiety into umbelliferone derivative **21** (Figure 11) resulted in significant *h*MAO-B blocking (IC_50_ = 10.32 μM). In the same studies, umbelliferone turned out to be less active than the tested compounds (*h*MAO-A IC_50_ = 18.08 μM and *h*MAO-B IC_50_ = 12.98 μM) [66].

It was proved that inhibition of D-amino acid oxidase (DAAO) may cause beneficial physiological effects on the central nervous system [67,68]. DAAO oxidizes D-amino acids (e.g.*,* the most significant substrate—D-serine) to the corresponding α-keto acids, ammonia and hydrogen peroxide. It has been reported that D-serine, which is present in the brain as a co-agonist of the NMDA receptor, can alleviate some symptoms of schizophrenia in humans. Therefore, the inhibition of the enzyme can be a potential therapeutic strategy for regulating the levels of D-amino acids in the brain and treating schizophrenia. Such beneficial effects on the central nervous system may provide a more comprehensive treatment for other neuropsychiatric disorders [67,68].

In 2022, Bester et al., as a result of their investigations, described the synthesis of 3-hydroxy-7-benzyloxy-2*H*-chromen-2-one (**22**) starting from 2,4-dihydroxybenzaldehyde, *N*-acetylglycine, and acetic anhydride (Figure 11) [69]. Compound **22** was identified as a potent, selective inhibitor of MAO-B (IC_50_ = 0.012 μM) and DAAO (IC_50_ = 1.86 μM). The results obtained were an improvement or comparable to those of the reference inhibitors: *coumarin* (MAO-B IC_50_ = 2.56 μM), *isatin* (MAO-B IC_50_ = 3.90 μM), and 3-methylpyrazole-5-carboxylic acid (DAAO IC_50_ = 1.88 μM). SAR analysis revealed that compound **22** inhibits MAO-B selectively due to the presence of a free hydroxyl group at the C-3 position and a bulky benzyloxy moiety at the C-7 position of the 1,2-benzopyrone ring. This 7-hydroxycoumarin derivative may represent a lead for the development of novel selective MAO-B inhibitors [69].

Overall, the abovementioned results suggest that umbelliferone-based compounds may be useful in the development of new neuropsychiatric drug candidates targeting monoamine oxidase and D-amino acid oxidase.

#### 4.2.2. Synthetic 7-Hydroxycoumarin-Based Compounds Targeting Serotonin Receptors

Balanced blocking of different types of serotonin and dopamine receptors in the central nervous system may reduce extrapyramidal side effects. This may extend the therapeutic effect to the negative symptoms of schizophrenia and affective disorders, i.e., mood disorders. This could be achieved through the use of atypical antipsychotic drugs, with a higher affinity for serotonin receptors than dopamine receptors which reduce overall side effects and increase the effectiveness of treatment [70].

**Figure 12:**
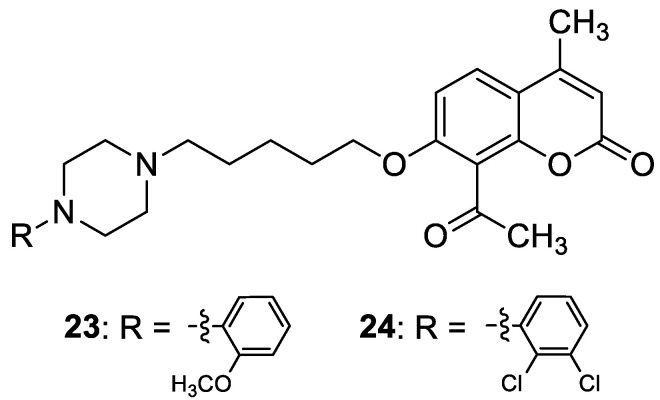
Chemical structures of 7-hydroxycoumarin-based compounds **23** and **24** targeting 5-HT receptors.

Recent studies have clearly demonstrated that umbelliferone-based compounds may interact with serotonin receptors. In 2021, among a series of 7-hydroxycoumarins bearing a piperazine moiety, 7-hydroxycoumarin derivatives **23** and **24** (Figure 12) showed high antagonistic activity against serotonin receptors [70].

In the paper, the authors claimed that the substitution pattern dictates the selectivity and affinity of tested compounds for 5-HT receptors. The structure–activity analysis showed that the presence of a five-carbon atom linker and 2-methoxyphenyl group attached to the piperazine moiety (compound **23**) was the most beneficial for 5-HT_1A_ antagonistic activity, whereas the (2,2-dichloro)piperazin-1-yl moiety is associated with a higher inhibition of the 5-HT_2A_ receptor (compound **24**). Compound **23** showed high antagonistic activity against the 5-HT_1A_ receptor (EC_50_ = 30.5 nM), although it was lower than reference 5-HT_1A_ antagonist *N*-[2-[4-(2-methoxyphenyl)-1-piperazinyl]ethyl]-*N*-2-pyridinyl-cyclohexanecarboxamide (WAY-100635, EC_50_ = 4.3 nM), whereas compound **24** exhibited moderate affinity for the 5-HT_2A_ receptor (*K*_i_ = 51 nM) compared to *ketanserine—*the known 5-HT_2A_ antagonist (*K_i_* = 3.6 nM). Overall, 7-hydroxycoumarin derivatives **23** and **24** have shown potential to be useful as therapeutic agents in the future [70].

**Figure 13:**
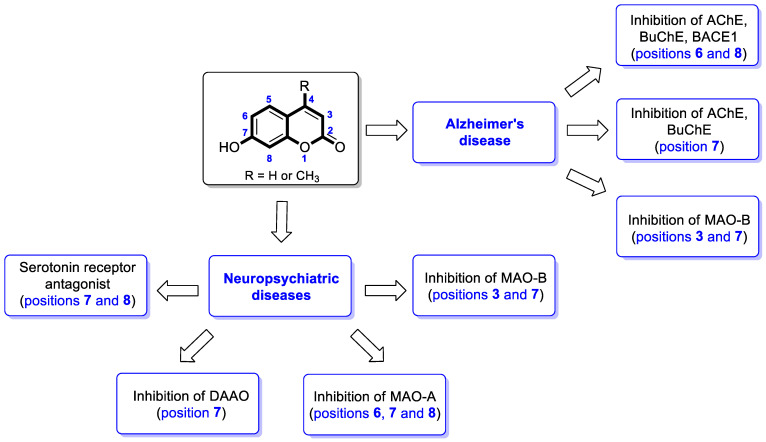
7-hydroxycoumarin’s substitution patterns and molecular targets in neurodegenerative and neuropsychiatric disorders.

In summary, the parent 7-hydroxycoumarin and its derivatives have shown important therapeutic effects in neurogenerative and neuropsychiatric disorders. The general 7-hydroxycoumarin substitution patterns and molecular targets for them in neurodegenerative and neuropsychiatric diseases are presented in Figure 13.

### 4.3. Antiepileptic Agents

Although there are many anticonvulsant agents in medical practice, their use is associated with possible drug interactions and severe side effects. Therefore, the development of novel antiepileptic drugs remains a vital area of the drug design process. Umbelliferone itself displays weak anticonvulsant activity [71].

Recently, it was confirmed that the administration of 7-hydroxycoumarin in combination with classical antiepileptic drugs such as *phenobarbital* and *sodium valproate* may benefit epilepsy treatment. It is well known that the substitution of coumarin at position 7 of the heterocyclic skeleton reduces its toxicity. Thus, 7-hydroxybenzopyran derivatives are an attractive starting point in the design of novel drugs containing this heterocyclic system. Umbelliferone was found to have neuroprotective properties in an excitotoxicity animal model of neurodegeneration [72].

**Figure 14:**
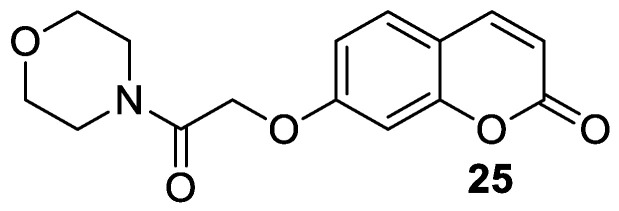
Chemical structure of 7-hydroxycoumarin-based compound **25** as an antiepileptic agent.

The antiepileptic effects of 7-hydroxycoumarin derivatives may be associated with the synergistic effect on γ-aminobutyric acid ionotropic receptors (GABA_A_). In the reaction of 7-hydroxycoumarin and 2-chloro-1-morpholinoethan-1-one, Yakovleva and collaborators synthesized umbelliferone derivative **25** (Figure 14) containing a morpholine-acetamide group at position C-7 [73]. Compound **25** showed pronounced antiepileptic activity in the *corazole*-GABA_A_ receptor antagonist convulsion test. The effectiveness of 7-hydroxycoumarin derivative **25** is associated with the morpholine ring, which has an optimal lipophilic-hydrophilic profile. The antiepileptic effect of derivative **25** at a dose of 200 mg/kg was comparable to that of reference valproic acid at the same dose. A further increase in the dose to 300 mg/kg led to an increase in the anticonvulsant activity of **25 [73]**.

## 5. Umbelliferone and 7-Hydroxycoumarin-Based Compounds as Antidiabetic Agents

Extracts of widely cultivated plants, such as *Musa* species (banana flower ethanolic extracts) containing umbelliferone, were identified as potential antidiabetic herbal remedies in the management of diabetes and associated complications. Isolated umbelliferone increased the activity of crucial enzymes involved in glucose utilization and the glycolytic activity of the liver in alloxan-induced diabetic rats [74]. The methanolic extracts of the annual plant *Bassia eriophora*, whose native range extends from the Eastern Mediterranean to Mongolia and the Arabian Peninsula, as well as the pure umbelliferone isolated from it have the potential to ameliorate *streptozocin* (STZ)- and high-fat diet (HFD)-induced damage to beta cells of Langerhans islets [75].

Su et al. claimed that umbelliferone (20 mg/kg and 40 mg/kg) may improve in vivo insulin resistance, which is regarded as the major factor predicting type 2 diabetes [76]. The beneficial mechanism is attributed to the phosphorylation of insulin receptors, insulin receptor substrate (IRS)-1, and the activation of glycogen synthase kinase-3β, phosphoinositide 3-kinase (PI3K), and protein kinase (Akt). This suggests that umbelliferone may be a potential therapeutic agent for the treatment of type 2 diabetes.

Umbelliferone was also reported to be effective in diabetic cardiomyopathy (DCM) by suppressing Janus kinase2 (JAK2) and the signal transducer and activator of the transcription signaling pathway (STAT3) [77]. Moreover, umbelliferone in a type 2 diabetic rat model at doses of 10 and 30 mg/kg decreased levels of glucose, glycated hemoglobin (HbA1c), tumor necrosis factor (TNF-α), and interleukin-6 (IL-6).

In this context, it should be mentioned that 6-formylumbelliferone (**14**, Figure 8)—an example of a rare hydroxycoumarin derivative found in nature—is effective at reducing glucose levels. In 2022, Md Yousof Ali was the first who reported its antidiabetic properties isolated from *Angelica decursiva*—a herb used in traditional Korean and Chinese medicine [78]. The antidiabetic effect of 6-formylumbelliferone (**14**) has been attributed to the blocking of enzymes that play a crucial role in diabetes mellitus type 2 including protein tyrosine phosphatase 1B (PTP1B) (IC_50_ = 1.13 μM), α-glucosidase (IC_50_ = 58.36 μM), and human recombinant aldose reductase (HRAR) (IC_50_ = 5.11 μM). Furthermore, this 7-hydroxycoumarin derivative showed promising antidiabetic potential inhibiting advanced glycation end-product (AGE) (IC_50_ = 2.15 μM) formation and improving insulin sensitivity by promoting the glucose uptake in insulin-resistant C2C12 muscle cells [78].

Despite numerous efforts to develop synthetic analogues of coumarins as potential drugs, not much research has been conducted on their antidiabetic activity.

**Figure 15:**
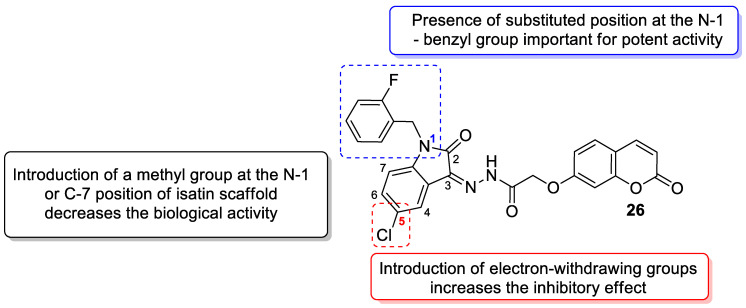
Chemical structure of 7-hydroxycoumarin-based compound **26** as an α-glucosidase inhibitor.

In 2017, Wang et al. reported novel coumarin-isatin derivatives as a novel class of α-glucosidase inhibitors [79]. The synthesized library of hybrids was composed of a 2-[(2-oxo-2*H*-chromen-7-yl)oxy]acetohydrazide fragment (7-hydroxycoumarin derivative) and a substituted isatin moiety. It was found that the introduction of electron-withdrawing groups at the C-5 position of the benzene ring of the isatin skeleton significantly increased their activity. Excellent inhibition was observed for compound **26** (Figure 15) with an IC_50_ value of 2.56 μM when compared to the reference drug—*acarbose* (IC_50_ = 817.38 μM). Further kinetic studies at different concentrations of compound **26** in the presence of *p*-nitrophenyl α-D-glucopyranose (*p*NPG) revealed that 7-hydroxycoumarin derivative **26** is a non-competitive inhibitor. Moreover, molecular docking simulations have confirmed a high binding affinity with *Saccharomyces cerevisiae* α-glucosidase through optimal hydrophobic and hydrogen interactions with the enzyme. Thus, compound **26** may serve as a leading structure in the development of novel α-glucosidase inhibitors that modulate postprandial hyperglycemia in type 2 diabetes [79].

## 6. Chemotherapeutic Activity

### 6.1. Antimicrobial Properties of Umbelliferone and 7-Hydroxycoumarin-Based Compounds

The search for 7-hydroxycoumarin-based compounds as antimicrobial agents has developed due to the rapid growth of the drug resistance of microbes. The antimicrobial activity of parent 7-hydroxycoumarin—umbelliferone of various origins—was reported several times in in vitro studies [14]. Pure 7-hydroxycoumarin showed activity against *Bacillus cereus* with a MIC and MBC value of 62.5 µg/mL. However, this coumarin exhibited rather moderate effectiveness against other enteropathogenic bacterial species of Gram-negative *Escherichia coli*, *Shigella sonnei,* and *Salmonella typhimurium*, as well as Gram-positive *Enterococcus faecalis* and *Staphylococcus aureus*. In addition, high concentrations were often required to inhibit the growth of most species tested (MIC = 500–1000 µg/mL) [14]. A few reports have also shown the modest activity of umbelliferone against *Pseudomonas aeruginosa* and *Staphylococcus epidermidis* (in the amount of 500 µg). On the other hand, 7-hydroxycoumarin was inactive in bacterial cultures of *Bacillus subtilis* and *Micrococcus luteus*, as well as fungal strains such as *Candica albicans* or *Aspergillus niger* [80,81,82].

Recently, the 7-hydroxycoumarin moiety was explored for its ability to inhibit biofilm formation by pathogenic bacterial strains. Firstly, the biofilm inhibitory properties of umbelliferone were shown at a concentration of 50 µg/mL against uropathogenic *E. coli* [83]. Similarly, umbelliferone exhibited significant antibiofilm activity (83%) against *methicillin*-resistant *S. epidermis* at a concentration of 500 µg/mL [84]. It was also found that the inhibitory effect on bacterial biofilm is not made through inhibiting the growth of bacteria, which is particularly desirable in the development of highly efficient antibiotic-resistant biofilm inhibitors [85].

In addition to antibiofilm activity, umbelliferone-treated cells exhibited enhanced susceptibility to various antibiotics against *P. aeruginosa.* Therefore, umbelliferone can be used in combination with other antibacterial agents to achieve an additive or synergistic therapeutic effect, which is an example of a combinatorial approach combining antibiofilm and antibiotic properties against human bacterial pathogens [86].

Moreover, in 2022, Supuran et al. reported for the first time the ability of umbelliferone to inhibit bacterial α-carbonic anhydrases (α-CAs) from the pathogens *Neisseria gonorrhoeae* (NgCAα) and *Vibrio cholerae* (VchCAα) in the medium micromolar range (*K_i_* = 77.1 µM and 68.5 µM, respectively) [87]. It is worth emphasizing that two human *h*CA isoforms, namely *h*CA I and *h*CA II, used for comparison in this study, were less prone to inhibition (*K_i_* = 263.5 µM and 690.6 µM). Overall, umbelliferone could be a starting point for further research into the development of 7-hydroxycoumarin-derived bacterial CA inhibitors as novel antibacterial agents.

#### 6.1.1. Synthetic 7-Hydroxycoumarin-Based Compounds as Antibacterial and Antifungal Agents

**Figure 16:**
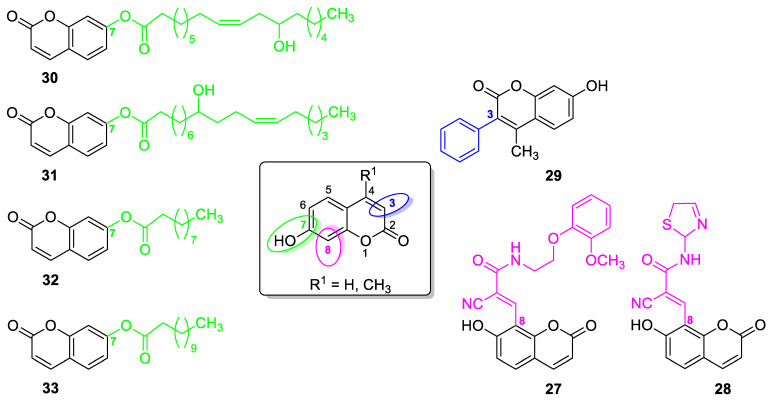
Chemical structures of 7-hydroxycoumarin-based compounds **27**–**33** as antibacterial and antifungal agents. The substitution pattern of the parent 7-hydroxycoumarin structure is indicated in color.

The design of novel synthetic 7-hydroxycoumarin-based compounds as antibacterial and antifungal agents was directed by a different substitution pattern of umbelliferone. However, the substitution at the C-8 position of the parent molecule was of great importance for biological activity. Thus, Manidhar Darla et al. described novel 8-substituted 7-hydroxycoumarins, which exhibited considerable activity against multi-drug-resistant bacteria *E. coli*, *S. aureus*, and *P. aeruginosa* as well as fungal strains *A. niger* and *C. albicans* [88]. Among them, compounds **27** and **28** (Figure 16) were selected as promising antibacterial and antifungal compounds because they were shown to be two-fold more potent than *norfloxacin* with MIC values of 4–6 µg/mL vs. MIC = 10 µg/mL against *E. coli*, *S. aureus*, and *P. aeruginosa*. Both of them were also three-fold more effective against *A. niger* and *C. albicans* than the first-line antifungal agent—*fluconazole* (MIC = 4–5 µg/mL vs. 12–14 µg/mL) [88].

The investigations in exploring 7-hydroxycoumarin derivatives as antimicrobial agents proved that modification of the coumarin nucleus at the C-3 position of the ring system was also important for future applications [89].

7-Hydroxycoumarin derivatives with an aliphatic or aryl moiety attached at the C-3 position of the coumarin skeleton directly or through various linkers were studied for their antimicrobial activity [89]. Among them, 7-hydroxy-4-methylcoumarin **29** with a phenyl moiety directly attached at the C-3 position of the coumarin ring system (Figure 16) was shown as a potential antibacterial agent against the *methicillin*-resistant *S. aureus* (MIC = 16 µg/mL) and the *vancomycin*-resistant *E. faecium* (MIC = 32 µg/mL).

In the early 1970s, it was revealed that the *O*-acylation of the parent coumarin increased the antimicrobial properties of 7-hydroxycoumarin [90]. Based on this approach, numerous *O*-acylated 7-hydroxycoumarins (7-hydroxycoumarin esters) with long-chain non-hydroxyl and hydroxyl fatty acid groups were expected to show increasing antimicrobial potency [91].

Preliminary screening of antimicrobial activity of the synthesized 7-*O*-coumarinyl alkenoates showed that compounds with a hydroxyl group in the alkenyl side chain possess greater activity than those with a non-hydroxyl carboxylic chain [91]. In particular, compounds **30** and **31** (Figure 16) displayed the highest activity against *B. subtilis*, *S. pyogenes*, *S. aureus*, and *E. coli* in the range of minimum inhibitory concentrations of 32–64 µg/mL comparable to the reference antibiotic—*chloramphenicol (*MIC *=* 32 µg/mL). Moreover, all compounds exhibited inhibitory activity against the fungi *C. albicans*, *C. parapsilosis,* and *Cryptococcus neoformans.* The aforementioned 7-hydroxycoumarin esters **30**–**31** displayed the strongest activity against *C. albicans* and they were not inferior to the reference drug—*fluconazole*. Especially, the inhibitory potency of compound **31** with MIC values of 2 µg/mL, 8 µg/mL, and 16 µg/mL against *C. albicans*, *C. parapsilosis,* and *C. neoformans,* respectively, was comparable to *fluconazole* (MIC = 1–8 µg/mL) [91].

Attempts at syntheses and testing of umbelliferone esters as antimicrobial agents led to the development of novel 7-hydroxycoumarin esters through the acylation reaction with different chain length vinyl esters catalyzed by the lipase Novozym 435 [92]. The bioactive assay revealed that compounds with alkyl chain lengths of 10 (7-decanoate umbelliferone ester **32**) and 12 carbon atoms (7-laurate umbelliferone ester **33**) (Figure 16) exert a powerful biological activity and may be considered as promising therapeutic candidates for the treatment of infectious disease. These compounds exhibited considerable efficacy against Gram-positive bacteria—*Staphylococcus* spp. including the *methicillin-* and *oxacillin*-resistant *S. aureus* strain with a MIC value of 1 mM—as well as Gram-negative bacteria, such as *P. aeruginosa* and *K. pneumoniae* with MIC values of 0.5 mM and 1 mM, respectively. In addition, they were able to inhibit clinical strains isolated from hospitalized patients with MIC values ranging from 0.5 mM to 1 mM, while other tested compounds and the parent 7-hydroxycoumarin were devoid of activities [92].

In the search for ideal antimicrobial drug candidates, the concept of molecular hybrids’ construction has been intensively explored over the last few years. According to this, hybrid molecules incorporating the 7-hydroxycoumarin nucleus with nitrogen-containing heterocyclic moieties occupy an important position in the development of antimicrobial agents with a broader antimicrobial spectrum. Moreover, recent studies on hybrid molecules have proved that the type of linker between coumarin and another pharmacophore skeleton affects biological efficacy [93,94,95].

**Figure 17:**
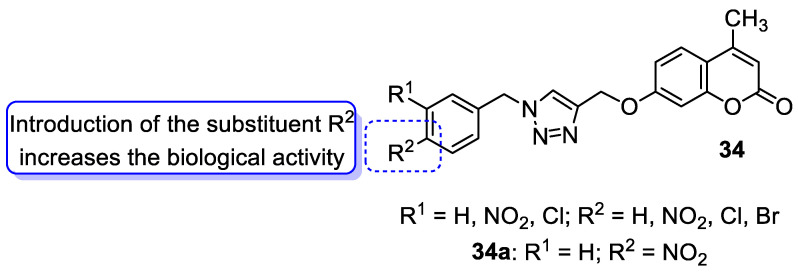
General structure of 7-hydroxycoumarin-1,2,3-triazole hybrids (**34**) as antibacterial and antifungal agents.

One of the highly explored coumarin hybrids is the combination of 7-hydroxycoumarin derivatives with the 1,2,3-triazole moiety using ether linker [95]. The study showed that coumarin-based hybrid compounds of general formula **34** (Figure 17) possess a potency to build good oral drug candidates for the treatment of infections especially in immunocompromised patients. Moreover, the structure–activity relationship analysis of this class of compounds revealed the importance of substitution of the phenyl moiety (R^1^, R^2^) in the hybrid molecules on their antimicrobial properties. Therefore, the introduction of a substituent at the *para* position of the aromatic ring (R^2^) resulted in more efficient biological activity than the *meta* substituent (R^1^). The most beneficial for biological activity was the introduction of a nitro group at the aromatic ring. Thus, novel coumarin hybrid **34a** (Figure 9) showed promising inhibitory potency against Gram-positive bacteria such as *M. luteus* and *B. cereus* with a MIC value of 4 µg/mL, and it was fourfold more potent than the standard drug—*ampicillin* (MIC = 16 µg/mL). In addition to this, the presence of a *para*-nitro group at the phenyl moiety resulted in increased efficacy against Gram-negative bacteria including *E. coli* and *P. fluorescens*; the above compound showed inhibitory activity at a relatively low concentration of 2 µg/mL in comparison with the most common clinical antibiotics—*ampicillin*, *chloramphenicol,* and *kanamycin* (MIC = 2–4 µg/mL). Moreover, 7-hydroxycoumarin-1,2,3-triazole hybrids substituted at the *para* position of the aromatic ring proved to be 2–4-fold more potent against the fungal strain *A. niger* (MIC = 4–8 µg/mL) compared to the standard drug—*miconazole* (MIC = 16 µg/mL) [95].

**Figure 18:**
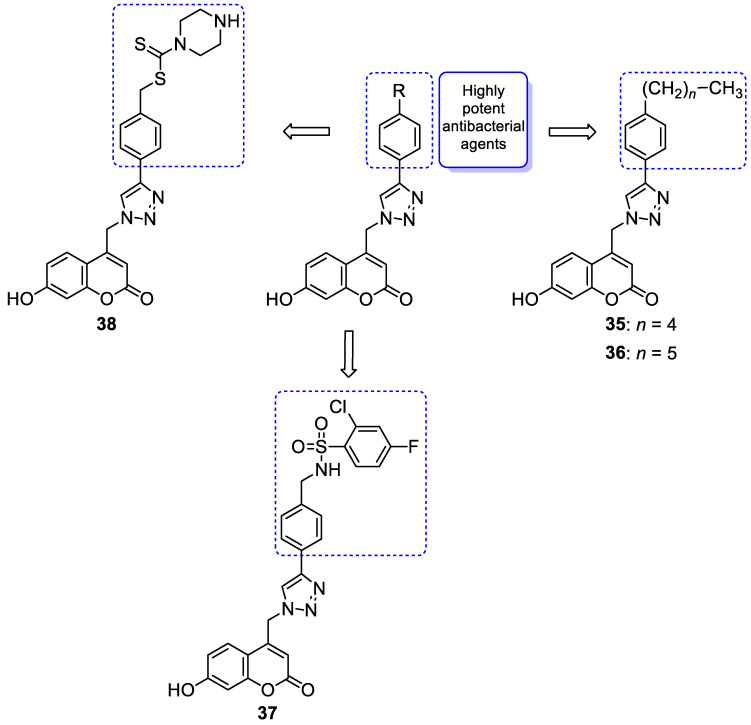
Chemical structures of 4-substituted 1,2,3-triazole-7-hydroxycoumarin hybrids **35**–**38** as antibacterial agents.

It is worth noting that numerous coumarin-1,2,3-triazole hybrids are promising candidates for the treatment of infections caused by multi-drug-resistant pathogens. A series of 7-hydroxycoumarin-based compounds linked to various 4-alkyl- or 4-aryl-1,2,3-triazole units through a methylene bridge at the C-4 position of the coumarin skeleton have been found as high-potential antibacterial agents [96]. SAR analysis indicates that the presence of *para*-alkylphenyl, 2-chloro-4-fluorobenzenesulfonamide, or dithiocarbamate substituents at the C-4 position of the triazole ring favors high selectivity towards *Enterococcus* species that are considered formidable pathogens. Hence, the selected hybrids **35**–**38** (Figure 18) were found to be superior in inhibiting the growth of clinically isolated *vancomycin*-resistant (VRA) *E. faecium* (MIC = 8–64 µg/mL), while the most common antibiotics, e.g., *ceftazidime* and *ciprofloxacin*, exhibited a lack of activity. Of special interest is hybrid **35** which was fourfold more potent than *ceftazidine* against VRA *E. faecium* and *E. faecalis* (MIC = 8 µg/mL and 64 µg/mL vs. MIC = 256 µg/mL) [96].

**Figure 19:**
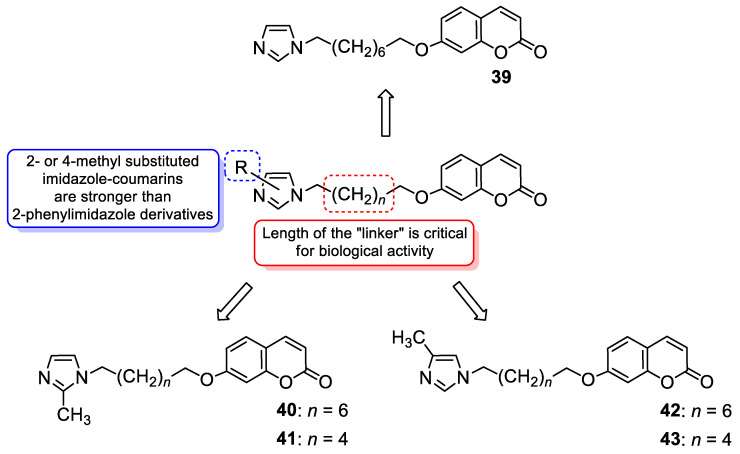
Chemical structures of 7-hydroxycoumarin-imidazole hybrids **39**–**43** as antibacterial agents targeting FabI and FabK.

With regard to the antimicrobial activity of coumarin-based hybrids, some studies have focused on the combination of the 7-hydroxycoumarin nucleus with the imidazole moiety. In 2018, Hu et al. described coumarin derivatives containing an imidazole core connected via an alkyl spacer to the 7-hydroxycoumarin scaffold as potential antibacterial agents targeting the type II bacterial fatty acid synthesis of the enoyl-acyl carrier protein reductases—the FabI and FabK steps [97]. Inhibition of FabI and FabK activity is a new strategy to solve bacterial drug resistance. The SAR study of this type of derivative revealed that the number of methylene units between coumarin and the imidazole skeleton as well as the substituent group at the imidazole ring are critical for both the antibacterial activity and the inhibitory effect against FabI and FabK. The optimal structure of the hybrid consists of eight linker carbon atoms to maximize the activity against Gram-positive *S. aureus* and *S. agalactiae* and Gram-negative *Flavobacterium columnare* bacteria. An expanded series of hybrids containing imidazole or 2- as well as 4-methyl substituted imidazole revealed a superiority to compounds with a 2-phenylimidazole moiety. Hence, compounds 39, 40, and 42 (Figure 19) displayed remarkable efficacy against *S. aureus*, *S. agalactiae,* and *F. columnare* (MIC = 2–16 µM and MBC = 8–128 µM) and significant FabK inhibitory activity (IC_50_ = 1.13–3.59 µM) [97].

Much attention has been paid to hybrid **40** (Figure 19), which showed antimicrobial activity against the three mentioned bacterial strains that was higher than the reference drug—*norfloxacin* (MIC and MBC values of 2–16 µM and 8–128 µM vs. MIC and MBC values of 32–64 µM and 64–128 µM)—and comparable or lower than *enrofloxacin* (MIC and MBC values of 2–16 µM and 8–128 µM vs. MIC and MBC values of 2–6 µM and 2–16 µM) [97]. In addition, compound **40** displayed the best FabK inhibition potency with an IC_50_ value of 1.13 µM. It is worth noting that compounds with six linker carbon atoms displayed a more definitely improved activity against *E. coli* than the other lengths of linkers. Thus, derivatives **41** and **43** (Figure 19) were found to exhibit high activity against *E. coli* (MIC = 8 µM and 16 µM, respectively, and MBC = 64 µM) and maintain favorable MIC and MBC values against *S. aureus*, *S. agalactiae,* and *F. columnare* (MIC = 16–32 µM, MBC = 32–256 µM) compared to *enrofloxacin* (MIC = 1–8 µM, MBC = 2–16 µM) and *norfloxacin* (MIC = 1–64 µM, MBC = 2–128 µM). Furthermore, compounds **41** and **43** showed pronounced FabI and FabK inhibitory properties with IC_50_ values of 1.20–1.35 µM and 3.44–3.55 µM, respectively. According to the data, hybrids **41** and **43** could serve as promising lead compounds for development of novel drug candidates with a broad spectrum of antibacterial activity acting through enoyl-acyl carrier protein reductase inhibition [97].

**Figure 20:**
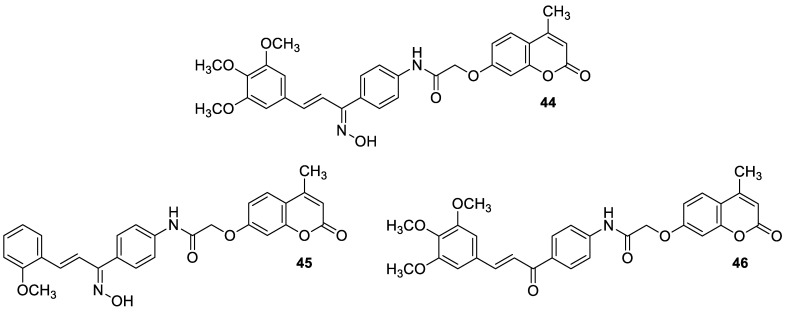
Chemical structures of 7-hydroxycoumarin-chalcone hybrids **44**–**46** as antibacterial agents.

Recently, the combination of 7-hydroxycoumarin derivatives with a chalcone moiety in one molecule linked through a simple oxyacetamide linker and their oxime-containing analogues has gained great interest [98]. This class of hybrid molecules constitutes an interesting group of compounds with potent antimicrobial activity against Gram-positive (*S. aureus* and *P. aeruginosa)* and Gram-negative (*E. coli* and *K. pneumoniae)* bacteria with MIC values in the range of 1.15–260 µg/mL; however, their exact mechanism of action has not been determined yet. Numerous hybrids proved to be most effective against cultured *S. aureus*, possessing comparable or even stronger effects than the reference drug—*levofloxacin* (MIC = 45 µg/mL). Among them, compounds **44** (MIC = 9.8 µg/mL) and **45** (MIC = 1.15 µg/mL) (Figure 20) were 4.6- and 39.1-fold more potent than the standard antibiotic. The structure–activity relationship analysis revealed that the incorporation of the oxime group into the structure of hybrids resulted in the enhancement of the antibacterial activity against Gram-positive *S. aureus* but caused a dramatic reduction in the potency against Gram-negative *E. coli* and *K. pneumoniae*. Thus, the compounds containing a ketone group were more efficient towards Gram-negative bacteria than their oxime analogues. In this line, hybrid **46** (Figure 20) turned out to be the most promising with the inhibitory potency MIC = 35.8 µg/mL and 9.6 µg/mL against Gram-positive (*S. aureus*) and Gram-negative (*E. coli*, *K. pneumonia*) bacterial strains [98].

**Figure 21:**
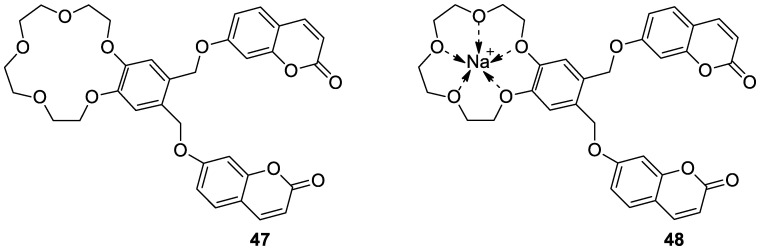
Chemical structures of 7-hydroxycoumarin-crown ether compounds **47** and **48** as antibacterial and antifungal agents.

Remarkable examples of potent antimicrobial agents are 7-hydroxycoumarin-substituted crown ether **47** and its sodium complex **48** (Figure 21) presented by Sahin Gül et al. [99]. The above compounds exhibited a good activity against opportunistic pathogens including Gram-positive bacteria *M. luteus*, *B. cereus,* and *S. aureus,* and they were equipotent towards Gram-negative bacteria such as *P. vulgaris* and *E. coli.* The antibacterial activity of these compounds was comparable to the effectiveness of the reference antibiotics: *ampicillin*, *nystatin*, *kanamycin*, *sulphamethoxazol,* and *amoxicillin*. On the other hand, hybrid **47** and its sodium complex **48** have been found to be more effective in inhibiting the growth of yeast *C. albicans* than standard drugs [99].

#### 6.1.2. Metal Complexes of 7-Hydroxycoumarin-Based Compounds as Antibacterial and Antifungal Agents

**Figure 22:**
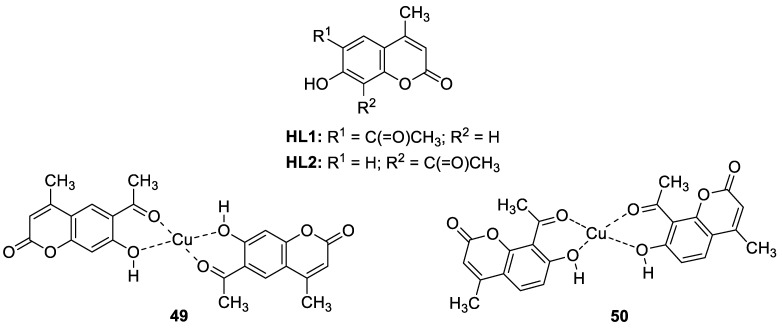
Chemical structures of ligands **HL1**, **HL2,** and their copper(II)-complexes **49** and **50** as antibacterial and antifungal agents.

Intending to enhance the antimicrobial activity of umbelliferone and its derivatives, researchers are opting for the preparation of their metal complexes. Among them, trioorganotin(IV) [100], Co(II), Ni(II), or Zn(II) [101] complexes of 7-hydroxycoumarin-derived ligands have shown enhanced in vitro antimicrobial activity compared to the parent ligands with low toxicity. Worth noting are also copper(II) complexes **49** and **50** with 6-acetyl-7-hydroxycoumarin **HL1** and 8-acetyl-7-hydroxy-4-methylcoumarin **HL2**, whose antimicrobial properties were evaluated against Gram-positive and Gram-negative bacterial strains as well as fungal strains (Figure 22) [102]. Investigation of biological activity revealed that these complexes exert improved potency in comparison to parent ligands against Gram-positive bacteria *S. aureus*, *B. subtilis*, and *B. cereus*, but they also gain activity towards other bacterial strains such as *S. epidermis*, *P. aeruginosa,* and *E. coli*. Additionally, the Cu(II) complex **50** with 8-acetyl-7-hydroxy-4-methylcoumarin was found to have a similar potency as an antifungal agent against *C. albicans* strains compared with *fluconazole* (MIC = 0.0375–0.075 mg/mL vs. MIC = 0.0125–0.256 mg/mL) [102].

The antibacterial activity has also been described for the Cu(II) complex derived from 8-formyl-7-hydroxy-4-methylcoumarin, which was invented as a promising pathogenic microorganism inhibitor by cleaving the supercoiled plasmid pBR322 DNA [101].

**Figure 23:**
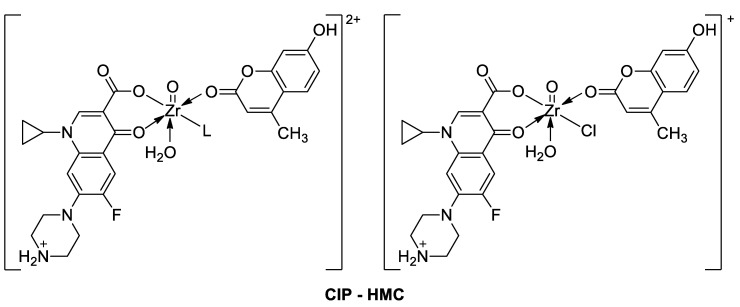
New Zr(IV) complexes formed from the interaction of *ciprofloxacin hydrochloride* and 7-hydroxy-4-methylcoumarin (**CIP-HMC**) (**L** = DMF, Py, and Et_3_N) as antibacterial agents.

Sadeek et al. demonstrated that compounds based on octahedral mixed-ligand complexes of Zr(IV) with *ciprofloxacin hydrochloride* as the primary ligand and 7-hydroxy-4-methylcoumarin as the secondary ligand with different coordination modes (**CIP-HMC**, Figure 23) possess promising biological activity against Gram-positive bacterial strains, including *B. subtilis* (MIC = 0.50–0.75 µg/mL) and *B. cereus* (MIC = 0.25–0.75 µg/mL), as well as Gram-negative bacterial strains, including *P. aeruginosa* (MIC = 0.50–1.0 µg/mL)*, K. pneumoniae* (MIC = 0.50–1.0 µg/mL), and *E. coli* (MIC = 0.50–1.0 µg/mL), compared to the free *ciprofloxacin* (**CP**: G + ve bacterial strains MIC = 0.50 µg/mL, G − ve bacterial strains MIC = 0.5–0.75 µg/mL) and 7-hydroxy-4-methylcoumarin (**HMC**: G + ve bacterial strains MIC = 0.25 µg/mL, G − ve bacterial strains MIC = 0.25–0.50 µg/mL). The chelation process accelerates the drug action, increasing the potency of both *ciprofloxacin* and coumarin molecules especially towards *B. subtilis* (diameter of inhibition zone: 48–66 mm vs. 18–26 mm) [103]. It should be mentioned that, in general, metal complexes are more active than their ligands and may serve as carriers to enhance the activity of ligands as the principal acting agents [101]. Thereby, these new Zr(IV) complexes build attractive molecules for further studies to determine the efficient concentrations of coumarin derivatives in the complexes and their mode of action [103].

### 6.2. Synthetic 7-Hydroxycoumarin-Based Compounds as Antituberculosis Agents

**Figure 24:**
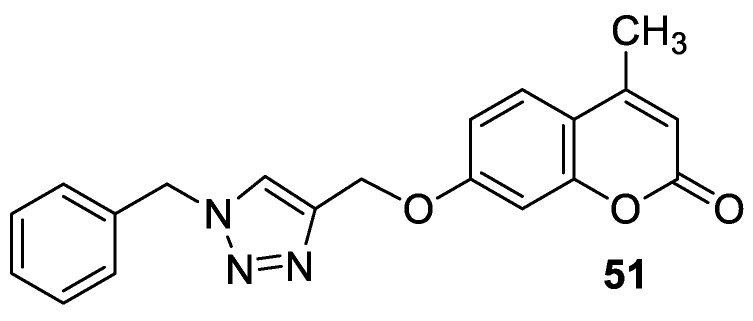
Chemical structure of 7-hydroxycoumarin **51** incorporating a triazole moiety as antitubercular agent.

The 7-Hydroxycoumarin skeleton has also been considered as a pharmacophore for searching for new antitubercular agents. Umbelliferone isolated from the whole plants of *Fatoua pilosa* exhibited potent activity against *Mycobacterium tuberculosis* H_37_Rv with a MIC value of 58.3 µg/mL [104]. Many coumarin-containing derivatives have been screened for their antitubercular properties. One study reported in recent years proved that 4-methyl-7-hydroxycoumarin-1,2,3-triazole hybrids with antibacterial activity (Figure 18) are a promising source of new mycobacterial cell wall-targeted candidates for the treatment of tuberculosis [96]. Some of the coumarin-based triazole derivatives exhibited improved efficacy against the *M. tuberculosis* H37Ra strain in comparison to the first-line drug—*pyrazinamide*—with IC_50_ values in the range from 1.8 µg/mL to 4.0 µg/mL vs. an IC_50_ value of 10 µg/mL. Among them, compound **51** (Figure 24) has been claimed to be the most potent antitubercular agent with an IC_50_ value of 1.8 µg/mL. Data of the molecular docking model showed that hybrid **51** interacts relatively more strongly with DprE1 (decaprenylphosphoryl-β-D-ribose-2′-epimerase), an enzyme essential for the biosynthesis of the mycobacterial cell wall, than other ligands. These results are in agreement with the observed antitubercular activity. It was suggested that the promising affinity towards the active site of the DprE1 enzyme may provide a molecular basis for new structure-based design efforts [96].

### 6.3. Synthetic 7-Hydroxycoumarin-Based Compounds as Antimalarial Agents

Malaria is an acute disease, transmitted by mosquitoes and caused by several protozoan *Plasmodium*, that can evolve rapidly and be lethal within days. Therefore, patients suspected of having an infection should be urgently diagnosed and, if confirmed, treated immediately. On the other hand, with the increasing drug resistance of malaria parasites, there is a need for new therapeutic agents and drugs, taking into account safety and improved dosing convenience [105].

**Figure 25:**
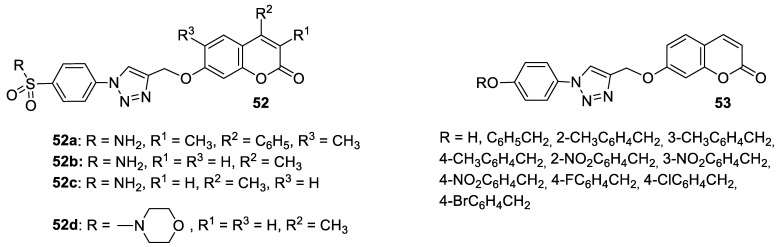
Novel 7-hydroxycoumarin-triazole hybrids **52** and **53** as antimalarial agents.

A series of sulfonamide-based coumarin-1,2,3-triazole conjugates as potential antimalarial agents have been developed [106]. Among them, hybrid **52** (Figure 25) displayed significant activity against the *P. falciparum* 3D7 strain, responsible for the most lethal form of malaria, at concentrations of IC_50_ < 10 µM. In comparison with a drug used in the prevention and treatment of malaria—*chloroquine* (IC_50_ = 0.066 µM)—the best result was found for compound **52b** with an IC_50_ value of 3.64 µM [106].

Worth mentioning are also coumarin-1,2,3-triazole hybrids of general formula **53**, previously described as antioxidant agents, which were effective against *P. falciparum* at concentrations of IC50 values ranging from 2.20 to 0.38 µg/mL (Figure 25) [43]. 7-[[1-[4-[(4-methylbenzyl)oxy]phenyl]-1*H*-1,2,3-triazol-4-yl]methoxy]-2*H*-chromen-2-one (53, R = 4-CH3C6H4CH2) has been claimed to possess encouraging antimalarial potential when compared to the standard drug—*quinine* (IC50 = 0.38 µg/mL vs. IC50 = 0.268 µg/mL).

### 6.4. Umbelliferone and 7-Hydroxycoumarin-Based Compounds as Antiviral Agents

Viral infections and their severe complications are a global public health concern. As a result of these epidemics, according to the World Health Organization, there are about 3 million to 5 million cases of severe illness every year and approximately 290 million to 650 million respiratory deaths every year. It has been estimated that 250,000 to 500,000 deaths are caused by the influenza virus each year [107]. Moreover, the influenza virus constantly mutates, giving rise to novel strains. Gradual transmission of the infection causes seasonal and pandemic influenza to spread over the population of the world. Thus, there is still a need to design more effective anti-influenza agents. One promising strategy is to develop small-molecule antiviral compounds that would protect against multiple strains.

Umbelliferone was claimed to possess antiviral activity which makes it suitable for the treatment of diseases caused by viruses [108].

Recently, umbelliferone present in Chinese mugwort (*Artemisia argyi*) showed promising activity against the entry of SARS-CoV-2 into cells, targeting proteins transmembrane serine protease 2 (TMPRSS2) and angiotensin-converting enzyme 2 (ACE2). As a result, 7-hydroxycoumarin suppressed the infection of ACE2-expressed HEK-293 T cells with lentiviral-based pseudo-particles (Vpp) expressing the wild-type and variants of the SARS-CoV-2 spike protein (SARS-CoV-2 S-Vpp). Moreover, it was found that oral administration with umbelliferone efficiently prevented the SARS-CoV-2 S-Vpp-induced inflammation in the lung tissues of BALB/c mice [109].

**Figure 26:**
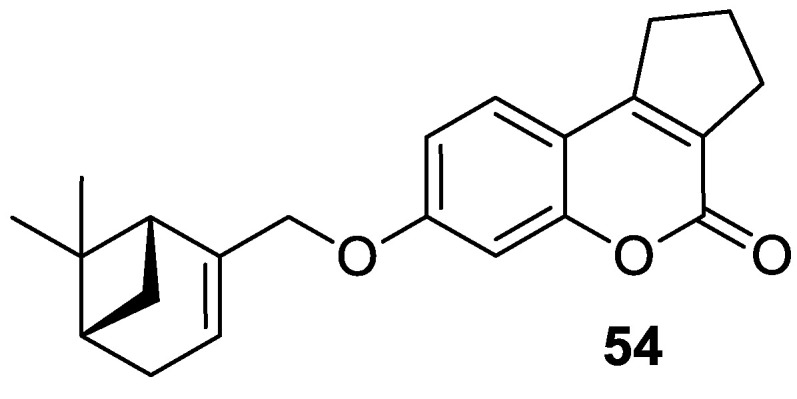
Chemical structure of 7-hydroxycoumarin-based compound **54** as an antiviral agent.

The 7-hydroxycoumarin-based compounds having the bicyclic pinane framework were effective in treating the influenza A virus. Of special interest is the derivative containing the (−)-myrtenol **54** (Figure 26) with a significant anti-influenza activity compared with the reference drug *rimantadine* (IC_50_ = 36 µM vs. IC_50_ = 9 µM) [110].

It was found that compound **54** exhibited the highest activity when added to the infected cell culture at the early stages of viral reproduction (1–2 h after infection). It has been suggested that the most likely targets of this molecule are the viral hemagglutinin or proton channel M2, a protein that leads to viral infection. Moreover, compound **54** is characterized by the highest selectivity index calculated as the ratio between the cytotoxicity and the active dose. Due to the promising activity (IC_50_ = 36 µM), low cytotoxicity (SI = 28), and high synthetic accessibility, compound **54** was claimed to possess the greatest potential and constitutes an important candidate for antiviral therapeutics [110].

Another class of novel agents suitable for preventing or treating infectious diseases are the aforementioned 7-hydroxycoumarins connected with a nitrogen-containing heterocycle by a methylene bridge [97]. Worth mentioning is derivative **41** containing a 2-methylimidazole ring and a six-membered linker depicted in Figure 19 which showed activity against the infectious hematopoietic necrosis virus (IHNV) [111]. Compound **41** significantly inhibits IHNV replication in EPC cells with an IC_50_ value of 2.53 µM and a CC_20_ value of 17.13 µM. Moreover, after treatment with 7-hydroxycoumarin derivative **41**, the cytopathic effect (CPE) of infected cells was decreased at 72 h. IHNV-infected cells treated with compound **41** maintained a normal spindle shape and kept a spherical shape with a clear edge, suggesting that the apoptosis can be blocked. Thus, derivative **41** may be regarded as a robust inhibitor of the IHN virus in an aqueous environment without overt cytotoxicity to host cells [111].

### 6.5. Umbelliferone and 7-Hydroxycoumarin-Based Compounds as Anticancer Agents

Umbelliferone has attracted considerable interest due to its anticancer activity. Preclinical progress indicates its usefulness in the future therapy of many solid tumors including oral epithelial carcinoma, colorectal, skin, prostate, breast, lung, or bladder cancer as well as central nervous system tumors [112,113,114]. Regardless of the type of cancer, umbelliferone activates mechanisms mediating cell cycle arrest, apoptosis (programmed cell death), or the inhibition of cancer migration and invasion. Moreover, research in recent years has provided the basis for a better understanding of its molecular mechanism of action in in vitro and in vivo studies [5].

Vijayalakshami et al. thoroughly investigated the effect of umbelliferone on oral epithelial carcinoma cells (KB) in a dose- and time-dependent manner [115]. The results suggested that the coumarin-mediated accumulation of reactive oxygen species (ROS) caused cell cycle arrest at the G0/G1 phase, depolarization of the mitochondrial membrane, and cell death via DNA damage.

Recent findings regarding anticancer activity of 7-hydroxycoumarin have also brought new perspectives in the therapy of highly malignant tumors, such as hepatocellular carcinoma (HCC). Yu et al. demonstrated for the first time the anticancer effect of umbelliferone on HepG2 cancer cells, involving the induction of apoptosis, cell cycle arrest, and DNA fragmentation [116]. In turn, Khunluck et al. investigated its inhibitory effect on the migration of cholangiocarcinoma (CCA) cells [117]. The authors found that umbelliferone could downregulate the expression of quinone oxidoreductase 1 (NQO1), the remarkable overexpression of which is correlated with a poor prognosis for many oncological patients. Thus, umbelliferone is a promising agent for CCA treatment; however, additional studies are required.

In recent years, the antitumor properties of umbelliferone in human lung carcinoma and renal carcinoma have also been better understood. Lopez-Gonzalez et al. proved that 7-hydroxycoumarin suppressed cell growth by arresting the cell cycle in the G1 phase in lung carcinoma cell lines and induced apoptosis in lung adenocarcinoma cells, but it was not related to intra-nucleosomal DNA fragmentation [118]. In fact, other studies have revealed that umbelliferone can inhibit the proliferation of lung adenocarcinoma cells (A427) by modulating the expression of proteins positively regulating the cell cycle—cyclin D1—and proteins involved in apoptosis such as Bcl-2 and Bax. Accordingly, 7-hydroxycoumarin caused a decrease in the level of cyclin D1 and Bcl-2, whereas an increase in Bax expression was observed in cultured cells [119,120]. These findings were in agreement with the results obtained after the umbelliferone treatment of human renal carcinoma cells. Moreover, it was shown that 7-hydroxycoumarin contributed to cell death by reducing p110γ protein expression [121].

Recent advances in research also proved the chemoprotective effect of the molecule in early-stage (LnCap) and late-stage prostate cancer (PC3) [122]. Umbelliferone treatment induced cell cycle arrest, caspase activation, and enhanced Bax expression in PC cells via NF-κB-independent pathways. Kim et al. showed the molecular mechanism of its antiproliferative activity in related diseases such as benign prostatic hyperplasia (BHP) [123]. Umbelliferone suppressed BHP cell proliferation by modulating the signal transducer and activator of transcription 3 (STAT3)/E2F transcriptor factor 1 (E2F1) axis. The treatment of cells with umbelliferone inhibited androgen receptor (AR) signaling-related markers and downregulated the overexpression of G1/S phase cell cycle-related markers. Advanced research also proved its ameliorative effects on prostatic hyperplasia in rat models [123].

Other investigations indicated that umbelliferone may offer a therapeutical strategy to overcome platinum drug resistance because it showed a selective cytotoxic effect against *cisplatin*-resistant ovarian cancer cells with significantly less activity on normal cells (IC_50_ = 12 µM vs. IC_50_ = 95 µM) [124]. These experiments confirmed observations reported by other authors that 7-hydroxycoumarin possesses the ability to induce the caspase-related apoptotic pathway. Additionally, cell cycle arrest at the G2/M stage has been demonstrated through the downregulation of regulatory proteins that promote mitotic entry. 

Umbelliferone has also been shown to be effective in the treatment of breast cancer, especially the highly aggressive, invasive triple-negative type. Nevertheless, the use of 7-hydroxycoumarin in combination with *piperine* caused the inhibition of a triple-negative breast cancer cell line (MDA-MB-231) to a greater extent in comparison to the application of coumarin alone (percent cell viability of 15.21 and 10.31, respectively) [125].

In turn, research conducted by Sumorek-Wiadro et al. has indicated that umbelliferone at a concentration of 200 μM may initiate programmed cell death in high-grade malignant gliomas, e.g., rapidly growing type anaplastic astrocytoma (MOGGCCM), and glioblastoma multiforme (T98G) in ca. 7% and 15% of cells, respectively [126,127]. On the other hand, the treatment of cells with 7-hydroxycoumarin and a potent anti-glioma drug—*temozolomide—*did not increase the pro-apoptotic potential of coumarin [126], whereas the co-incubation with *sorafenib*, a Raf kinase inhibitor, was more effective, causing apoptosis in up to 17% of cells [127].

Considering the current research and future anticancer therapy, construction of smart mesoporous silica nanoparticles (MSNs) of the compound targeting malignant tumors is highly demanding [128]. The study proved that the use of MSNs loaded with umbelliferone and functionalized with pH-sensitive polyacrylic acid (PPA) and folic acid (FA) resulted in the effective delivery of coumarin through the binding of FA to folate receptors on cancer cells. In fact, the synthesized nanohybrid (Umbe@MSN-PAA-FA) caused oxidative stress and mitochondrial damage leading to cellular apoptosis in human breast MCF-7 cancer cells with the overexpression of folate receptors. It should be noticed that the in vitro study revealed a higher anticancer activity of Umbe@MSN-PAA-FA compared with free umbelliferone in MCF-7 cells at equivalent drug concentrations. Furthermore, the advanced studies proved Umbe@MSN-PAA-FA’s efficacy in reducing the tumor growth in tumor-bearing mice as well as the non-toxicity towards the vital organs [128].

Overall, umbelliferone is an important platform in the search for new potential anticancer agents with a diverse mechanism of pharmacological action.

#### 6.5.1. Synthetic 7-Hydroxycoumarin-Based Compounds as Histone Deacetylase (HDAC) Inhibitors

In addition to genetic alterations, aberrant epigenetic modifications of gene expression may also be involved in tumor initiation and progression. Histone acetylation plays a pivotal role in the epigenetic regulation of gene transcription and expression through chromatin modification. The level of this process is balanced under physiological conditions by both histone acetyltransferases (HATs) and histone deacetylases (HDACs) [129]. HDACs were found to be overexpressed in various cancer types such as prostate, ovarian, breast, and colon cancer or leukemia, with the fateful result of gene transcription and expression deregulation influencing a variety of cellular functions, namely, proliferation and differentiation, angiogenesis, metastasis, cell death, autophagy, and metabolism [130]. Inhibition of HDACs exerts a number of anticancer effects, including the induction of cell cycle arrest, apoptosis, and blocking angiogenesis or metastasis. Thus, HDACs are viable therapeutic targets for the treatment of cancer.

7-Hydroxycoumarin-based HDACs inhibitors show huge structural diversity. However, the general pharmacophore consists of substituted 7-hydroxycoumarin combined with 2-aminobenzamide or hydroxamic acid via a hydrophobic linker.

**Figure 27:**
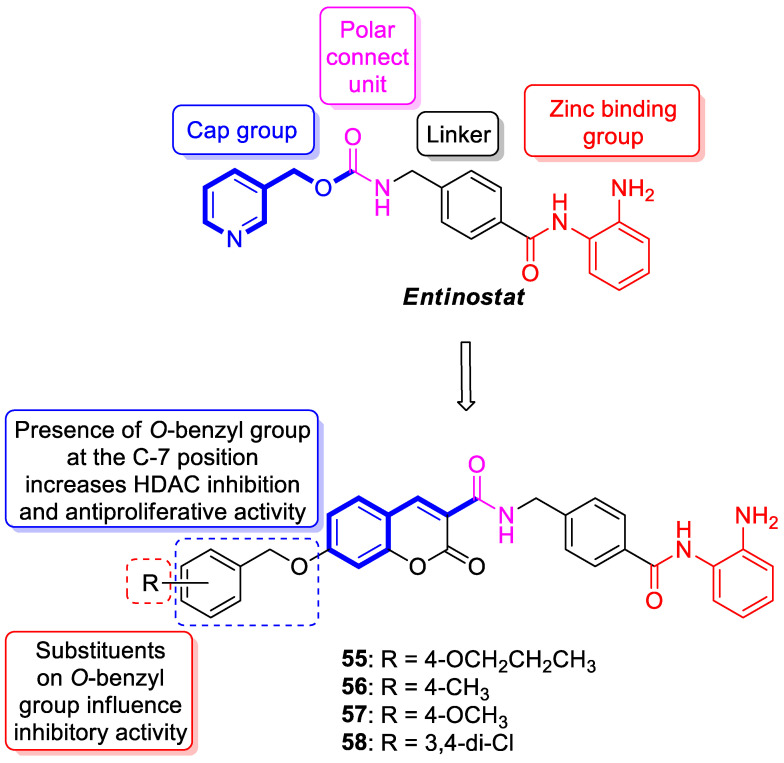
Chemical structures of 7-hydroxycoumarin-based benzamides **55**–**58** as HDAC1 inhibitors with anticancer properties.

Firstly, coumarin-based compounds as potent histone deacetylase inhibitors and anticancer agents were developed based on the structural modification of the selective class 1 HDAC inhibitor *entinostat* (Figure 27). In this approach, Abdizadeh et al. described the activity of novel HDAC inhibitors bearing 7-*O*-substituted coumarin carboxamide instead of the benzyl carbamate moiety of *entinostat* [131]. Numerous designed compounds (Figure 27) exhibited significant antiproliferative activity with IC_50_ values in the range of 0.27 µM to 61.87 µM against various cancer cells, especially colon (HCT116) and ovarian cancer (A2780) cells, as well as lung (A549), prostate (PC3), breast cancer (MCF7), and leukemia (HL60) cells. The structure–activity relationship study enabled the determination of coumarin substitution to evoke the target biological effect in cancer cells. It was identified that the chain length of the *O*-alkyl group at the C-7 position of the coumarin ring system had a significant effect on the potency; a bulky alkoxy substituent was preferable. On the other hand, the introduction of the *O*-benzyl group into the C-7 position of the coumarin moiety resulted in an increase in the HDAC inhibition potency as well as the antiproliferative activity in cancer cells. Additionally, the variety and position of substituents on the *O*-benzyl group were also important for the inhibitory activity. Within the series, the most potent 7-hydroxycoumarin derivative against tested tumor cell lines, derivative **55** (Figure 27) (IC_50_ = 1.69–16.6 µM), showed higher antiproliferative properties towards the HCT116, A549, and HL60 cell lines than the known clinically studied *entinostat* (IC_50_ = 0.27–3.14 µM vs. IC_50_ = 2.03–4.53 µM). Moreover, this compound was found to be the most potent HDAC inhibitor with IC_50_ values of 0.25 µM and 2.06 µM in the HCT116 and A2780 cell lines, respectively, and its inhibitory potency was greater than that of the reference drug (IC_50_ = 1.96 µM and 3.15 µM) [131].

**Figure 28:**
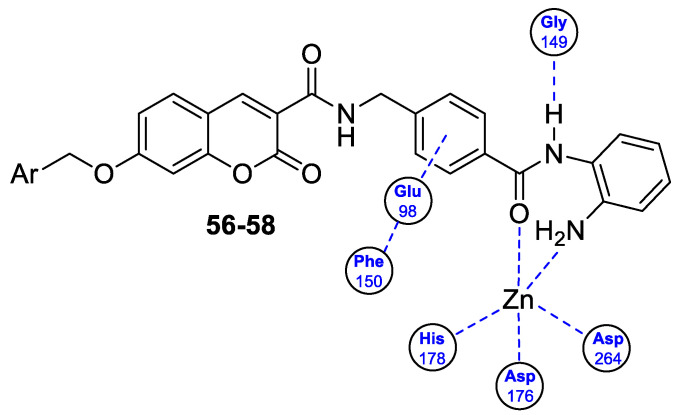
A 2D representation of the interaction between compounds 56–58 in the crystal structure of HDAC1.

Furthermore, compounds **56**–**58** (Figure 27) also displayed promising cytotoxicity effects on human cancerous cells (IC_50_ = 0.53–48.86 µM) and enzymatic inhibitory HDAC activity (IC_50_ = 0.80–5.41 µM) with unique HDAC1 isoform selectivity (IC_50_ = 0.47–0.87 µM) comparable to *entinostat* (IC_50_ = 0.41 µM). Molecular docking studies of the mentioned coumarins showed that these compounds interact with the active site of HDCA1 through the coordination of the zinc ion, strong hydrophobic interactions, and the formation of the hydrogen bond (Figure 28). It should be noted that the tested compounds did not exhibit significant toxic effects on normal HUVEC cell lines, which makes them promising candidates for developing new anticancer therapeutics [131].

**Figure 29:**
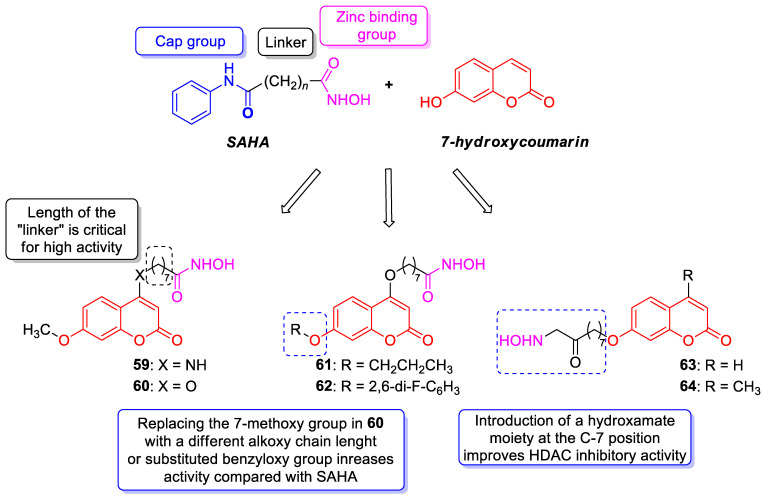
Chemical structures of 7-hydroxycoumarin-based hydroxamate derivatives **59***–***64** as HDAC1 inhibitors with anticancer properties.

The promising results of coumarin-based benzamides as potent HDAC inhibitors with anticancer activity encouraged the design of coumarins containing a hydroxamate moiety as HDAC inhibitors based on the structure of an FDA-approved deacetylase inhibitor for the treatment of cutaneous T-cell lymphoma—*vorinostat*, also known as suberoylanilide hydroxamic acid (*SAHA*, Figure 29) [132]. The designed compounds possessed a hydroxamic acid group linked via a spacer (CH_2_)_n_ to the C-4 position of the coumarin scaffold (Figure 29). SAR analysis proved that the HDAC1 inhibitory activity of novel compounds was linker-length-dependent and revealed that seven carbon spacers were sufficient for high activity. Thus, compounds **59** and **60** (Figure 29) were claimed to possess excellent inhibitory potency against HDAC1 (IC_50_ = 0.24 nM and 1.85 nM, respectively) [132].

In addition, coumarin **59** was nearly 90 times more active than *SAHA* against HDAC1 (IC_50_ = 0.24 nM vs. 21.10 nM). Preliminary docking studies proved the high specificity in binding of the coumarin-based compound **59** with the HDAC1 isoform. Moreover, compound **59** exhibited strong anticancer activity against the human lung adenocarcinoma A549 cell line and human cervical HeLa cancer cells with IC_50_ values of 1.96 µM and 1.31 µM, respectively, whereas compound **60** was more potent against the A549 cell line, demonstrating an inhibitory effect at a concentration of IC_50_ = 0.56 µM. These results indicate the stronger ability of the tested compounds to inhibit the growth of the mentioned tumor cells than *SAHA* with IC_50_ values of 2.63 and 2.86 µM [132].

According to the obtained results, coumarin-based hydroxamate **60** was considered as the lead structure to explore more coumarin-based HDAC inhibitors with better activities [133]. The modification of structure **60** consisted in replacing the methoxy group at the C-7 position of the coumarin core with a different alkoxy chain length or substituted benzyloxy group (Figure 29). A series of novel compounds were evaluated in vitro for their HDAC inhibitory activities. In general, the synthesized compounds were more active than the reference drug *SAHA*, and the alkoxy-substituted coumarins showed stronger inhibitory potency than the benzyloxy-substituted analogues. Furthermore, it was revealed that the potency of the alkoxy-substituted compounds improved with the appropriate elongation of the chain length, while the inhibitory effect of the benzyloxy-substituted ones depended significantly on the nature and position of the substituent at the benzyloxy group. Among them, 2-methoxyethoxy-substituted analogue **61** (Figure 29) was found to be the most potent HDAC1 inhibitor (IC_50_ = 0.30 nM) with significant growth inhibition against human MDA-MB-231, H157, and A549 cancer cell lines (IC_50_ = 0.36–2.79 µM), even better than *SAHA* (IC_50_ = 0.36–2.79 µM). Molecular docking proved the high binding potency of compound **61.** Hydrogen bonds, hydrophobic interactions, and zinc-coordinated interactions were proposed to explain its high affinity to the active site of the enzyme. The study also assessed the effect of derivative **61** on the highly metastatic human breast cancer cells (MDA-MB-231). In fact, it displayed antimetastatic and antiproliferative activities, arresting MDA-MB-231 cells at the G2/M phase and inducing cell apoptosis. Worth noting is also difluorobenzyloxy-substituted analogue **62** (Figure 29), described as a highly potent HDAC1 inhibitor (IC_50_ = 0.50 nM) with promising antiproliferative properties towards the MDA-MB-231, H157, and A549 cancer cell lines with IC_50_ values in the range of 1.95 µM to 7.58 µM [133].

Exploring various novel *SAHA* analogues, it was evidenced that the introduction of a hydroxamate moiety at the C-7 position of the 7-hydroxycoumarin skeleton could effectively improve HDAC inhibitory activity. Thus, novel target compounds **63**–**64** (Figure 29) displayed higher inhibitory effects against HDAC1 than *SAHA* (IC_50_ = 6.88 nM and 8.71 nM vs. IC_50_ = 21.10 nM) [132].

**Figure 30:**
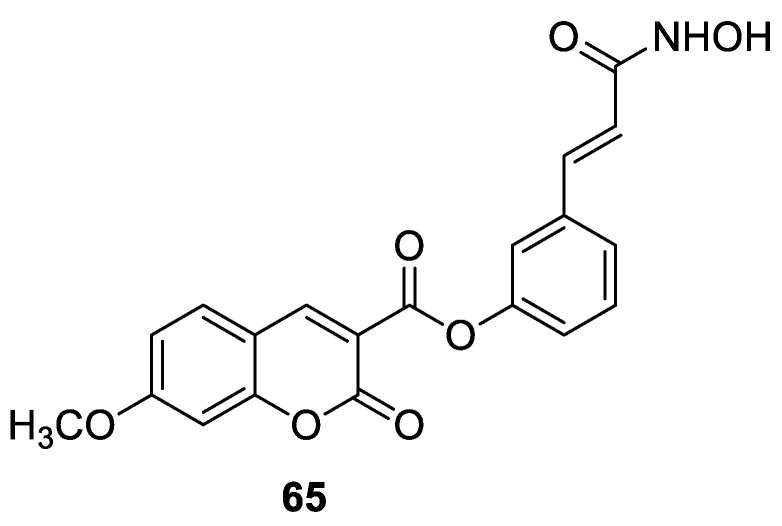
Chemical structure of 7-hydroxycoumarin-3-carboxylic-based *N*-hydroxycinnamide derivative **65** as an HDAC inhibitor with anticancer properties.

A series of 7-hydroxycoumarin-3-carboxylic-based *N*-hydroxycinnamide derivatives have also been described as histone deacetylase inhibitors with anticancer activity [134]. Among them, 7-hydroxycoumarin derivative **65** depicted in Figure 30 was identified as the most potent HDAC inhibitor (IC_50_ = 0.32 μM) with 26-fold selectivity for the HDAC1 isoform over the HDAC6 one (IC_50_ = 0.19 µM vs. 4.98 µM). These results were better than those of *SAHA* (HDAC IC_50_ = 0.48 µM; HDAC isoform selectivity: HDAC1 IC_50_ = 0.23 µM vs. HDAC6 IC_50_ = 0.22 µM) [134].

The molecular docking study revealed that this compound fits well into the active site of HDAC1 compared to the known HDAC inhibitor LBH589. On the other hand, its low affinity for HDAC6 was associated with the coumarin moiety, which changed its binding orientation upon contact with the active site of the enzyme. Furthermore, compound **65** exhibited broad and significant anticancer activity (IC_50_ = 6.91–13.32 µM) compared to *SAHA* (IC_50_ = 2.11–4.09 μM). The most sensitive cell line was found to be HeLa cancer cells (IC_50_ = 6.91 µM). The obtained results revealed that coumarin **65** is a promising candidate for the further development of novel HDAC inhibitors for anticancer therapy [134].

#### 6.5.2. Synthetic 7-Hydroxycoumarin-Based Compounds as Androgen Receptor (AR) Antagonists

**Figure 31:**
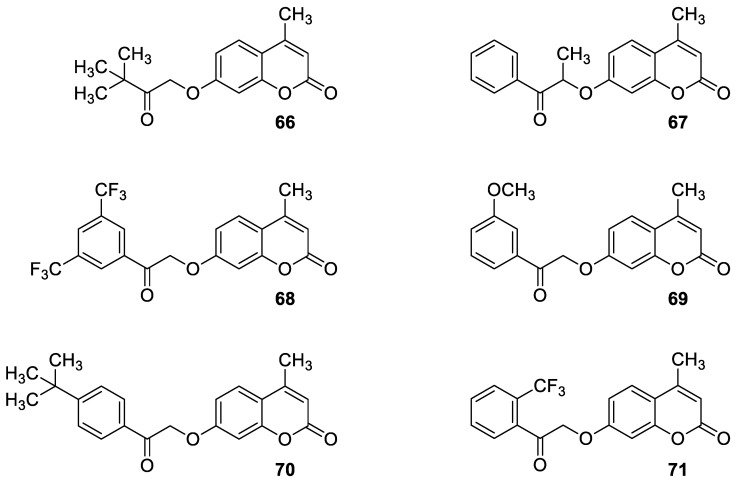
Chemical structures of 7-hydroxycoumarin-based compounds **66**–**71** as AR antagonists with anticancer properties.

Anticancer therapy may utilize androgen receptor (AR) signaling pathway inhibition, which has been implicated in the carcinogenesis and metastasis of hormone-related tumors, e.g., prostate and breast cancer. Driven by the need to search for unique AR antagonists, the in silico screening of small-molecule libraries of 7-substituted umbelliferone derivatives was applied [135]. Using a combined virtual protocol, two molecules, **66** and **67,** were identified (Figure 31) that interact with AR in a unique manner and act as pure AR antagonists [136]. Both of these chemotypes represented by structures **66** and **67** feature a 4-methyl-7-hydroxycoumarin core containing a β-keto-ether group at the C-7 position of the coumarin moiety. Unlike the clinically used AR antagonists—*bicalutamide*, *flutamide,* or *nilutamide*—both compounds **66** and **67** inhibit ARs in cellular models of hormone-refractory disease (IC_50_ = 3.4 μM and 5.1 μM, respectively) including those with mutant ARs (W741C, T877A) and wild-type AR overexpression [137,138].

**Figure 32:**
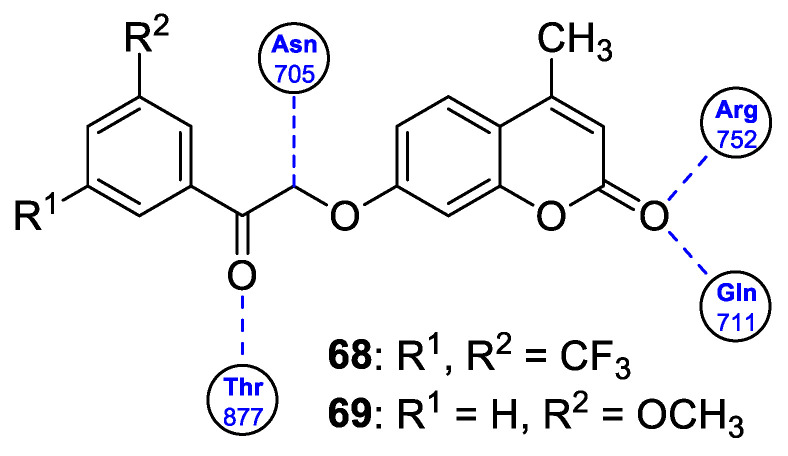
The 2D putative binding modes of compounds 68 and 69 inside the antagonistic hAR-LBD showing hydrogen bond interactions with key amino acids: Arg752, Gln711, Thr877, and Asn705.

Based on these findings, a novel series of umbelliferone derivatives varying in the terminal aromatic group of the ketone linkage at the C-7 position of the coumarin scaffold was designed and evaluated in in vitro studies for antiproliferative activity against the prostate 22Rv1 and breast MCF-7 cancer cell lines [138]. Within the series, compounds **68** and **69** (Figure 31) displayed remarkable antiproliferative activity against human prostate (22Rv1) and breast (MCF-7) cancer cells with IC_50_ values in the ranges of 0.93–22.27 μM and 0.47–43.21 μM. In turn, coumarins **70** and **71** (Figure 31) were superior in inhibiting the growth of 22Rv1 cells (IC_50_ = 22.27 μM and 20.37 μM, respectively) in comparison to clinically used drugs, including second-generation AR antagonist *enzalutamide* (IC_50_ = 31.76 μM) [138]. However, particular attention has been paid to compounds **68** and **69** with significant inhibitory effects on the growth of prostate cancer cells at low concentrations of IC_50_ = 0.93 μM and 8.41 μM, respectively. Interestingly, both analogues **68** and **69** were found to be more effective against breast cancer cells (IC_50_ = 0.47 μM and 2.21 μM, respectively). Molecular docking studies indicated the binding of these compounds in the human AR ligand-binding domain (Figure 32). It was assumed that the binding mode of compound **68** via H-bond interactions with the Arg 752 residue provides high activity, making it almost 50-fold more potent than *bicalutamide* and 30-fold more potent than *enzalutamide* [138].

#### 6.5.3. Synthetic 7-Hydroxycoumarin-Based Compounds as Inhibitors of the PIK3/Akt Signaling Pathway

The current work is more often focused on the design and study of novel 7-hydroxycoumarin-based compounds acting as inhibitors of the phosphatidylinositol-3-kinase (PI3K)/protein kinase B (Akt) signaling network. It is one of the most frequently dysregulated signaling pathways in the pathogenesis of breast cancer, associated with tumor initiation, survival, and invasion [139,140]. Oncogenic activation of this pathway is attributed to the mutation of genes encoding PI3K subunits—PIK3CA (p110α) and PIK3CB (p110β) [141,142]. On the other hand, as the role of this signaling network in cancer cells’ immunomodulation is better understood, Akt hyperactivation is also associated with the escape of cancer cells from immune recognition [143].

Recent investigations proved that 7-hydroxycoumarin-based compounds could display great therapeutic efficacy in breast cancer by targeting the PI3K/Akt signaling pathway [144]. These findings are highly encouraging in the context of the spread of resistance to current anticancer therapy.

**Figure 33:**
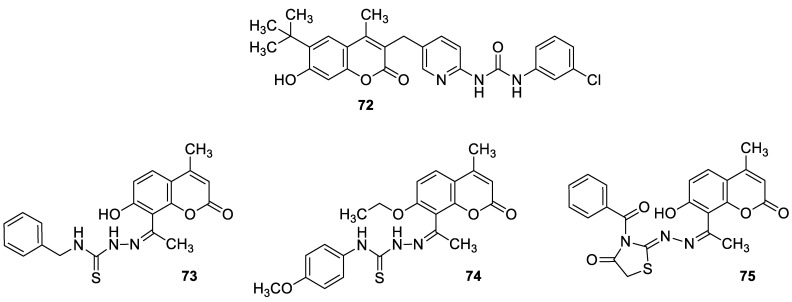
Chemical structures of 7-hydroxycoumarin-based compounds **72**–**75** as inhibitors of the PI3K/Akt signaling pathway with anticancer properties.

Novel synthesized compounds bearing a pyridinylurea substituent attached to the coumarin core at the C-3 position were expected to show favorable interactions with the hinge region of PI3Ks through the formation of the critical hydrogen bond with the backbone residue of valine [144]. Molecular structure analysis revealed that the nature of the substituent and its position on the distal aryl ring may have a great influence on the interactions with the ATP-binding pocket and differential potency. Preliminary in vitro screening selected compound **72** (Figure 33) as the most promising candidate with considerable growth inhibitory effects on human cancer cell lines, including lung carcinoma A549, breast carcinoma MCF-7, leukemia K562, and cervical carcinoma HeLa (IC_50_ values ranging from 2.17 µM to 7.13 µM), that was able to inhibit 84.1% of PIK3K activity. In addition, compound **72** turned out to be a selective inhibitor of PI3Kα/β/δ isoforms (PI3Kα/β/δ IC_50_ = 5.28–12.02 µM vs. PI3Kγ IC_50_ > 50 µM). Moreover, coumarin **72** was found to effectively block Akt phosphorylation in a concentration- and time-dependent manner as well as induce cell apoptosis mediated via the PI3K/Akt signal pathway with the cleavage of caspase-3 [144].

In turn, Abdelnaby et al. investigated dual PI3K/Akt-acting hybrids bearing 7-hydroxycoumarin derivatives and a thiosemicarbazone moiety or its cyclic form, a thiazolidin-4-one ring, attached at the C-8 position of the coumarin ring [145]. Several of the synthesized compounds exhibited comparable or improved cytotoxicity against the breast MCF-7 cancer cell line compared with standard drug *5-fluorouracil* (5-FU) (IC_50_ = 1.03–26.41 µM vs. IC_50_ = 27.81 µM). Within the coumarin-thiosemicarbazone series, compound **73** (Figure 33) displayed significant efficacy with an IC_50_ value of 5.13 µM, while hybrid **74** (Figure 33) was even 23-fold more potent than 5-FU (IC_50_ = 1.21 µM vs. IC_50_ = 27.81 µM) and demonstrated an excellent safety profile with a good selectivity index (SI = 16.61 vs. SI = 1.3) [145].

**Figure 34:**
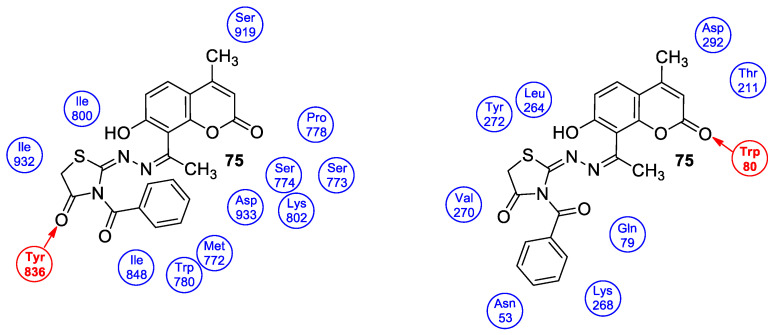
Compound **75** in the active site of PI3K (**left**) and Akt-1 (**right**).

The structure–activity analysis of this class of compounds revealed that the cyclization strategy and substitution pattern of the thiazolidine ring are important for the anticancer activity [145]. Thus, within the coumarin-thiazolidine series, a cyclic analogue of benzoyl derivative **75** depicted in Figure 33 was found to induce the most remarkable cytotoxic effect against MCF-7 cells at a low concentration of IC_50_ = 1.03 µM in comparison to the non-cyclic analogue (IC_50_ = 47.32 µM). Furthermore, the SI value for compound **75** was 9.24, showing a preferential effect on target cancer cells compared with 5-FU (SI = 1.30). The enzyme inhibition assay revealed the targeting of hybrid **75** to the PI3K-α/Akt-1 axis, whereas the results of the molecular docking study showed the pivotal role of Tyr836 in the binding of PI3K to compound **75** and Trp80 in the binding of Akt-1 to ligand **75** (Figure 34).

Moreover, the ability of compound **75** to modulate anti-apoptotic cyclin D1 was evidenced by decreased protein expression upon exposure to **75**. It was concluded that the observed antitumor efficacy in the MCF-7 cell line of novel coumarin **75** suggested its potential to evolve as a promising anticancer drug [145].

#### 6.5.4. Monoterpene-Coumarin Hybrids as Tyrosyl-DNA Phosphodiesterase 1 (Tdp1) Inhibitors

An interesting therapeutically useful anticancer strategy consists in inhibiting tyrosyl-DNA phosphodiesterase 1 (Tdp1). The Tdp1 enzyme plays a crucial role in the removal of DNA damage resulting from DNA-topoisomerase 1 (Top1) inhibition with Top1 poisons as well as some other chemotherapeutical drug-induced DNA damages [146,147]. It is well known that Tdp1 is involved in the development of tumor resistance to Top1 inhibitors [148,149]. On the other hand, suppression of Tdp1 activity may increase the sensitivity of tumor cells to Top1 inhibitors, potentiating their effects. Therefore, the Tdp1 enzyme is a promising target in cancer drug design [150,151].

**Figure 35:**
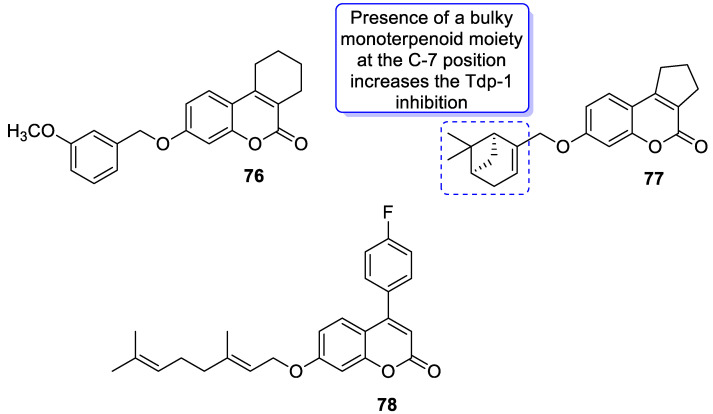
Monoterpene-coumarin hybrids **76**–**78** as Tdp1 inhibitors with anticancer properties.

The screening approach along with the oligonucleotide-based fluorescence assay have been successfully applied to identify 3-methoxybenzyl-7-hydroxycoumarin **76** annulated with the cyclohexane ring (Figure 35) as a new structural type of Tdp 1 inhibitor with an IC_50_ value of 4.93 µM [152].

The structural optimization revealed that the replacement of the phenyl group with a bulky monoterpenoid moiety at the C-7 position of the coumarin core could increase inhibitor potency. Thus, 7-hydroxycoumarin **77** (Figure 35) containing a monoterpene substituent at the 7-hydroxy group was found to show Tdp-1 inhibition at a low concentration of IC_50_ = 0.675 µM. Interestingly, compound **77** exhibited negligible cytotoxicity (CC_50_ > 100 µM) when tested against human cancer cells; however, it significantly enhanced the cytotoxic activity of the Top1 inhibitor—*camptothecin*—in cancer cells. These findings prompt further development of potential Tdp1 7-hydroxycoumarin-based inhibitors [152].

Novel hybrids bearing a 4-aryl-7-hydroxycoumarin core and monoterpenoid moieties were designed as potential tumor sensitizers for currently used antitumor drugs [153]. The synthesized compounds emerged as potent Tdp1 inhibitors with IC_50_ values in the submicromolar range. Of these, monoterpene-arylcoumarin hybrid **78** presented in Figure 35 was selected for in vivo studies using the Marine Krebs-2 carcinoma model, which revealed its synergistic effect with a clinically important Topo1 inhibitor—*topotecan*—against Krebs-2 carcinoma. Additionally, the novel hybrid **78** exhibited a high probability of good oral bioavailability that makes it a highly promising candidate for further development [153].

#### 6.5.5. Synthetic 7-Hydroxycoumarin-Based Compounds as Carbonic Anhydrase (CA) Inhibitors

In recent years, carbonic anhydrases (CAs) have gained attention as a potential target in anticancer drug development. Carbonic anhydrase is a family of ubiquitous zinc enzymes that play a crucial role in regulating the pH in various tissues and organs in the human body. In humans, the CA enzymes exist in 15 isoforms that vary by localization and activity. CAs catalyze the reversible conversion of carbon dioxide (CO_2_) and water (H_2_O) into bicarbonate (HCO_3_^−^) and protons (H^+^) [154]. This enzymatic activity is essential for maintaining the acid–base balance in the body. While carbonic anhydrases have physiological functions, they have also been explored as potential targets in anticancer drug development. Carbonic anhydrase isoforms IX and XII are highly overexpressed in hypoxic solid tumors, with limited presence in normal cells. Their overexpression contributes to tumor survival and metastasis, promoting chemoresistance to weaker anticancer drugs. Their presence in hypoxic tumors makes them an attractive drug target for hypoxic tumors and metastatic hypoxic tumors [155,156,157].

Compounds containing a coumarin ring system constitute a potent and relatively new class of carbonic anhydrase inhibitors. It was found that the benzopyrone ring undergoes hydrolysis of the lactone moiety mediated by the esterase activity of carbonic anhydrase. Due to this unique mechanism of inhibition of CAs, coumarin-based compounds may be classified as ‘prodrug-inhibitors’ [158]. Moreover, studies have shown that umbelliferone and its 7-hydroxy-substituted derivatives may selectively inhibit carbonic anhydrases IX and XII over I and II [159,160].

**Figure 36:**
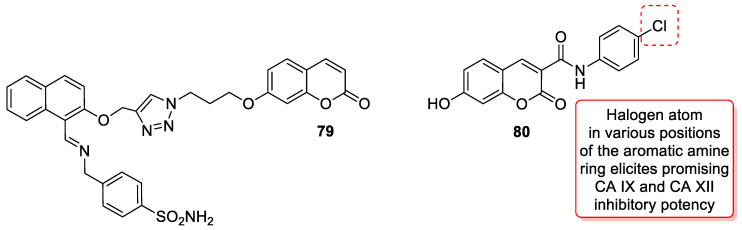
Chemical structures of 7-hydroxycoumarin-based compounds **79** and **80** as CA-IX and CA-XII inhibitors with anticancer properties.

Recently, the search for antitumor drugs led to the discovery of novel carbonic anhydrase inhibitors represented by a 7-hydroxycoumarin derivative containing primary sulfonamide moiety **79** (Figure 36) [161]. It has been demonstrated that compound **79** has a selective antiproliferation effect on the colorectal HT-29 cancer cell line, which has a high CA IX expression under ambient air (IC_50_ = 17.01 µM for HT-29, IC_50_ = 118.73 µM for embryonic kidney cell line HEK293T compared to the standard drug *doxorubicine* with IC_50_ values of 5.38 and 1.051 µM, respectively). Compound **79** inhibits the proliferation and migration of HT-29 cells in a dose-dependent manner and acts as an inducer of apoptosis. It was found that 7-hydroxycoumarin **79** suppresses the expression of CA IX and CA XII proteins in vitro in HT-29 cells; the *K*_i_ value calculated for CA IX equals 45.5 nM. According to these findings, **79** is able to block cellular proliferation in human colon cancer cells by specifically targeting the expression of CA IX and CA XII in these cells. This suggests that compound **79** may be considered as a potential therapeutic agent for the treatment of human colon cancer [161].

In 2019, among a series of novel 7-hydroxycoumarin-3-carboxamides, Thacker et al. reported *N*-(4-chlorophenyl)-7-hydroxy-2-oxo-2*H*-chromene-3-carboxamide (**80**) (Figure 36) as exhibiting a submicromolar potency against tumor-associated, transmembrane-bound carbonic anhydrases *h*CA IX and *h*CA XII [162]. The concentration value required to produce half the maximum enzyme inhibition *K*_i_ for the designed small-molecule inhibitor **80** was calculated as 0.2 μM. The cytosolic isoforms *h*CA I and II were not inhibited by the tested compound (*K*_i_ > 100 μM). With a view to shed more light on the interaction of 7-hydroxycoumarin derivative **80** with the binding site of the carbonic anhydrases *h*CA IX (PDB 3IAI) and *h*CA XII (PDB 4WW8), docking studies have been performed. Compound **80** displayed interactions mainly with Thr199 and Gln92 of *h*CA IX. In the case of *h*CA XII, 7-hydroxycoumarin **80** interacts with Zn301, Thr198, and Asn64, which may contribute to its potency towards *h*CA XII. It was concluded that compound **80**, specifically designed to target the transmembrane tumor-associated isoforms *h*CA IX and *h*CA XII, can potentially serve as a lead structure for the development of novel anticancer therapeutic agents [162].

**Figure 37:**
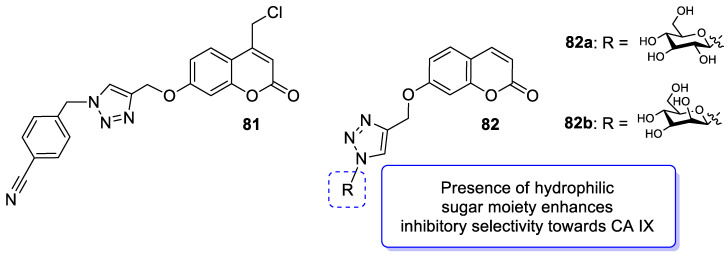
Chemical structures of 4-substituted 1,2,3-7-hydroxycoumarin hybrids **81** and **82** as selective CA IX inhibitors with anticancer properties.

An interesting class of hybrid compounds—4-chloromethyl-7-hydroxycoumarins linked via the 1,2,3-triazole ring—has been reported to be effective as selective inhibitors of the tumor-associated isoform hCA IX [163]. The lowest in vitro inhibition constant was achieved by compound **81** (Figure 37) containing a *para*-substituted cyano group at the benzene ring (*K*_i_ = 32.7 nM); the calculated *K*_i_ constant for *acetazolamide* (AAZ) as a standard CA inhibitor equals 25.8 nM. Hence, 7-hydroxycoumarin derivative **81** could be taken as a lead compound for the further design and development of selective and potent hCA IX inhibitors [163].

It was suggested that the introduction of the sugar moiety into the chemical structure of the designed carbonic anhydrase inhibitors can lead to a significant enhancement of their activity [164,165,166]. In this context, of great importance for the development of new chemotherapeutic agents are carbohydrate-based 7-hydroxycoumarin derivatives (**82**) (Figure 37) comprising a biocompatible covalent heterocyclic linker designed by Chu et al. [167]. Among the synthesized compounds, 7-[(1-β-D-glucopyranosyl-1*H*-1,2,3-triazol-4-yl)methoxy]-2*H*-chromen-2-one (**82a**) and its analogue containing mannose—7-[(1-β-D-mannopyranosyl-1*H*-1,2,3-triazol-4-yl)methoxy]-2*H*-chromen-2-one (**82b**, Figure 37)—showed the most potent *h*CA IX inhibitory activities with IC_50_ values of 11 nM and 15 nM, respectively. The increase in the inhibitory potency towards isoform CA IX may be attributed to the matching of the hydrophilic sugar moiety with the hydrophilic half of the active site of the enzyme. On the other hand, there is strong interaction between the umbelliferone core and the hydrophobic half of the active site. Moreover, the rigid 1,2,3-triazole linker—a bioisostere of the amide group, displaying a moderate dipole character—possesses high hydrogen bonding capability and stability under physiological conditions, as well as tolerance to metabolic processes. Compound **82a** (Figure 37, IC_50_ = 11 nM) displayed higher inhibitory activity towards CA IX than the reference drug *acetazolamide* (IC_50_ = 30 nM), reducing tumor cell viability and the extracellular acidification in the HT-29 and MDA-MB-231 cancer cell lines. These results supported by docking studies suggest that 7-hydroxycoumarin derivative **82a** may serve as a lead structure for developing anticancer medications [167].

The presented research results indicated that the discovery of 7-hydroxycoumarin-based compounds as inhibitors of carbonic anhydrases IX and XII may stimulate the search for new drugs with specific effects in cancer therapy.

#### 6.5.6. Synthetic 7-Hydroxycoumarin-Based Compounds as Cyclooxygenase-2 (COX-2) and 5-Lipoxygenas (5-LOX) Inhibitors

The development of novel antitumor drugs based on the inhibition of cyclooxygenase-2 (COX-2) has been an important part of antitumor drug development, because COX has proven to be a promising target in the design of antitumor agents. There is a growing understanding that several inflammatory mediators, such as cytokines, chemokines, and growth factors, may promote cancer formation and progression by controlling the tumor microenvironment. Furthermore, COX-2, an enzyme nearly undetectable in most normal cells or tissues, is upregulated in tumors at all stages [168,169,170,171].

Similar to COX-2, lipoxygenases (LOXs) are pro-inflammatory enzymes associated with arachidonic acid (AA) cascade. In this pathway, AA is transformed into hydroxyeicosatetraenoic acids derivatives (HETEs) and leukotrienes (LTs), which play a major key role in the development and progression of human cancers as a result of LOX activation. In particular, the overexpression of 5-LOX has been shown to have significant effects on the cell cycle, preventing apoptosis and stimulating angiogenesis. A growing body of evidence suggests that some types of cancer are related to higher levels of 5-LOX and its main product, leukotriene B4 (LTB4). Furthermore, COXs and LOXs promote tumor growth and dominate inflammation [172,173]. In light of the similarity of the action of these two enzymes, the dual inhibition of both enzymes may provide more efficient and safer agents for the treatment of human cancers, thereby becoming more effective and safer. Thus, a promising strategy in cancer therapy may involve the inhibition of both COX-2 and LOX.

**Figure 38:**
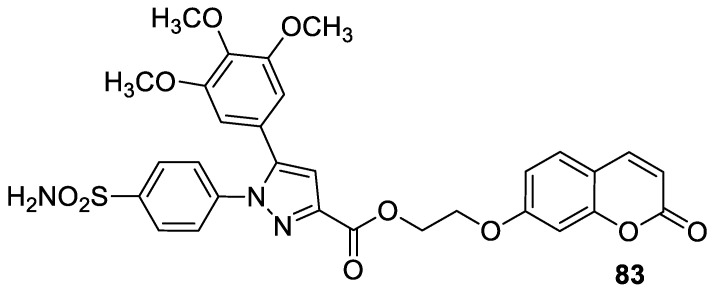
Chemical structure of COX-2 and 5-LOX dual inhibitor **83** as an anticancer agent.

In this line, Shen et al. designed a novel COX-2 and 5-LOX dual inhibitor composed of the 1-(4-sulfamoylphenyl)-5-(3,4,5-trimethoxyphenyl)-1*H*-pyrazole and 7-hydroxycoumarin moieties [174]. A high selectivity level has been observed for compound **83** (Figure 38) towards enzyme subtypes based on its IC_50_ values of 0.23 µM for COX-2 and 0.87 µM for 5-LOX, making the tested compound superior to *celecoxib* as a positive control for COX-2 (IC_50_ = 0.41 µM) and *zileuton* for 5-LOX (IC_50_ = 1.35 µM). 7-hydroxycoumarin derivative **83** was tested against four different cancer cell lines (A549: human lung cell line, HeLa: human cervix cell line, SMMC-7721: human liver cell line, HT-29: human colorectal cell line) and one non-cancer cell line (293T: human kidney epithelial cells) exhibiting the most potent activity against A549 cancer cells with an IC_50_ value of 4.48 µM, compared with the positive control *celecoxib* (IC_50_ = 7.68 µM). It was suggested that the presence of three methoxy groups in the structure of the 1*H*-pyrazole-containing 7-hydroxycoumarin **83** would increase hydrogen bonding, thus affecting the affinity of the compound with the protein. Further investigation confirmed that derivative **83** could induce human non-small cell lung cancer A549 cells into apoptosis and arrest the cell cycle at the G2 phase in a dose-dependent manner [174].

#### 6.5.7. Metal Complexes of 7-Hydroxycoumarin-Based Compounds as Anticancer Agents

As reported in the scientific literature, 7-hydroxycoumarin derivatives were used as promising ligands coordinated with different metal ions for developing novel, more potent, and safer metallodrugs for anticancer therapy.

The extracellular protein kinase (ERK)/mitogen activated protein kinase (MAPK) signaling pathway plays a crucial role in regulating cancer cell growth, apoptosis, and metastasis. Therefore, the ERK/MAPK pathway is the subject of intense research leading to the development of inhibitors for the treatment of cancer [175].

**Figure 39:**
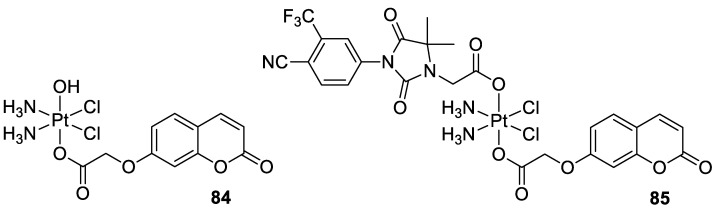
Chemical structures of 7-hydroxycoumarin-based Pt(IV) complexes **84** and **85** as anticancer agents.

In 2018, Hua et al. described cou-platin (**84**, Figure 39) composed of 7-hydroxycoumarin and a platinum(IV) moiety derived from *cisplatin* as more potent towards a variety of cancer cells than *cisplatin* (IC_50_ = 0.08–2.46 µM vs. IC_50_ = 1.86–9.34 µM) [176]. The mechanistic studies with the use of human colon carcinoma HCT116 cells revealed that new Pt-binding molecule **84** is able to inhibit cancer cell growth via activation of cell apoptosis and inhibition of the ERK/MAPK signaling pathway. Since the significant anticancer effect of cou-platin **84** was observed in cell cultures, this compound was subjected to in vivo tests in a mouse model, which proved that **84** at a dose equimolar to 9 mg/kg of *cisplatin*, could efficiently inhibit the growth of HCT116 cells xenografted in nude mice with less toxicity than the references drugs—*cisplatin* and *oxaliplatin*. These findings suggest the potential of cou-platin **84** to improve efficacy and reduce toxicity compared to current *cisplatin* therapies [176].

In turn, Pt(IV) conjugate **85** (Figure 39), composed of an AR-binding nonsteroidal cyanonilutamide unit, 7-hydroxycoumarin, and *cisplatin* moiety, represents the AR antagonist intended for castration-resistant prostate cancer treatment [177]. Compound **85** possess potent AR binding affinity (IC_50_ = 7.58 μM) and satisfactory antagonistic activity against androgen receptors (70% inhibition at 10 μM). Moreover, molecule **85** displayed excellent cytotoxic effects towards human prostate adenocarcinoma cells (IC_50_ = 1.02 µM), while the ligands were inactive at a concentration of IC_50_ < 50 µM. Notably, hybrid **85** was 9.6-fold more potent than *cisplatin* and 46.5-fold more potent than nonsteroidal antiandrogen *bicalutamide*. Further analysis of the mechanism of its action revealed that compound **85** could achieve an apoptosis rate of 83%, much superior to that of *cisplatin*. It significantly arrested the cell cycle at the S phase and dramatically increased apoptosis [177].

**Figure 40:**
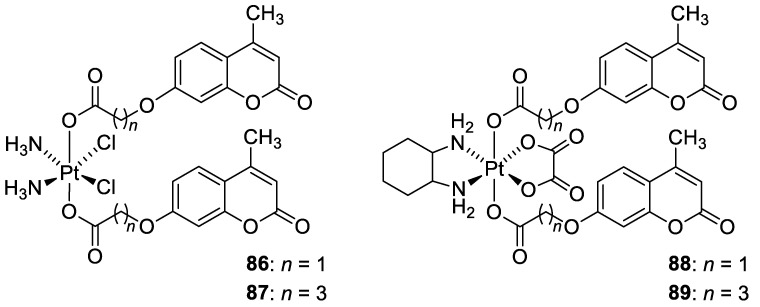
Chemical structures of 7-hydroxycoumarin-based Pt(IV) complexes **86**–**89** as anticancer agents with a bi-functional mechanism of biological action.

Malignancy-related inflammation is one of the factors contributing to the development and spread of many types of cancer; thus, Wang et al. have examined novel bifunctional platinum(IV) compounds with 7-hydroxycoumarin ligands arranged in axial positions, which were designed to have both antitumor and anti-inflammatory properties (**86**–**89**, Figure 40) [178]. It has been found that the coumarin platinum(IV) complex **88** inhibits the human *rh*COX-2 enzyme activity from 20.1% to 65.8% in a concentration-dependent manner. By releasing an appropriate coumaric acid derivative, compound **88** reduces tumor-associated inflammation. It was suggested that the reduction of platinum(IV) complexes **86**–**89** into appropriate platinum(II) compounds may also occur in tumor tissues, causing the DNA to be damaged in the tumor cells. As a result of this, it may be concluded that coumarin platinum(VI) complexes exhibit a bi-functional mechanism of action [178].

**Figure 41:**
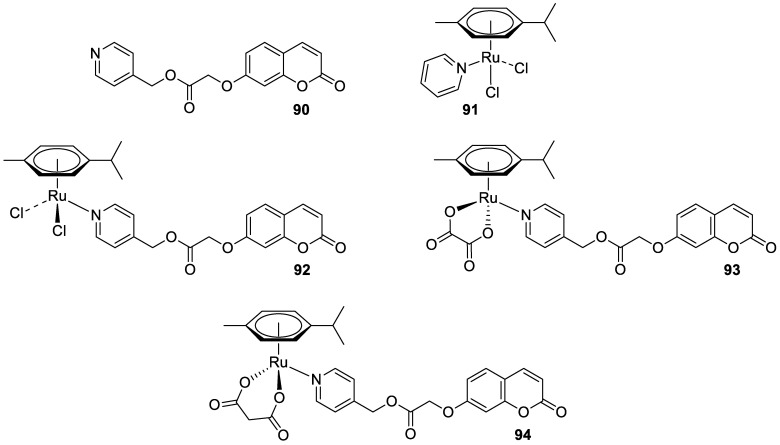
Chemical structures of ligand **90** and Ru(II) complexes **91**–**94** as anticancer agents.

Ruthenium(II) complexes have attracted attention as promising alternative candidates to platinum complexes for anticancer therapy due to their lower toxicity and higher selectivity. Because they have been widely investigated either as single anticancer compounds or in combination with other cytotoxic agents, it was expected that a synergistic pharmacological effect could be achieved by the introduction of the 7-hydroxycoumarin scaffold into therapeutically important Ru(II)-arene complexes [179,180,181]. In fact, organometallic Ru(II)-arene compounds **92**–**94** containing a 7-hydroxycoumarin group showed stronger cytotoxic effects on cancer cell lines HCT-116 (colorectal cancer), HepG-2 (hepatocellur carcinoma), and A549 (non-small cell lung cancer) than ligand **90** and non-functionalized complex **91** (IC_50_ = 65.6–161.4 µM vs. IC_50_ > 500 µM) (Figure 41). On the other hand, the cytotoxicity of complexes **92**–**94** was lower than that of *cisplatin* (IC_50_ = 13.6–16.5 µM) [182]. Complex **92** (Figure 41) was found to be the most potent with IC_50_ values ranging from 65.6 µM to 78.7 µM. Further study indicated that newly synthesized complexes can induce cascade cell apoptosis through the mitochondrial pathway including activating Bax-induced cytochrome C release, which results in caspase-3 activation. Additional analysis showed that Ru(II) complexes **92**–**94** may also prevent MEK1 and ERK1 phosphorylation, leading to apoptosis via inhibition of the ERK signal pathway. Therefore, they appear to be promising candidates for the development of anticancer therapeutics that can improve antitumor effects by acting on multiple targets [182].

Over the last few years, *N*-heterocyclic carbene (NHC) gold(I) complexes have been described as inhibitors of selenoenzyme thioredoxin reductase (TrxR) with antiproliferative properties in cancer cell lines [183].

**Figure 42:**
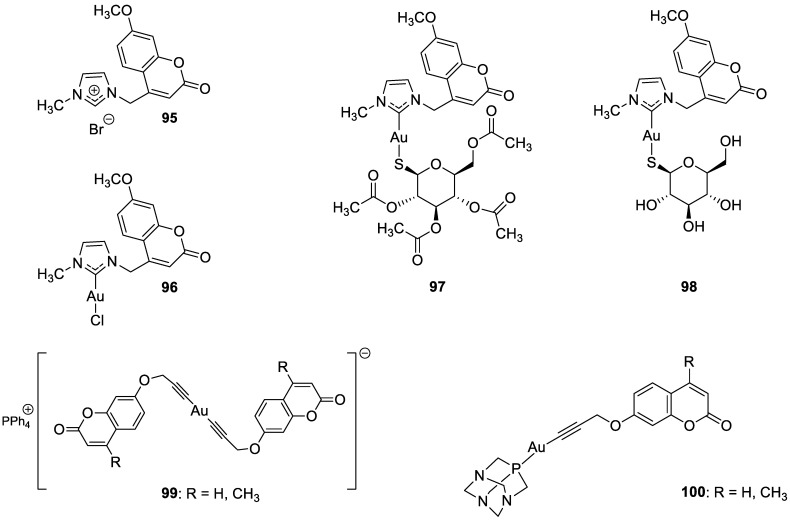
Chemical structures of ligand **95** and 7-hydroxycoumarin-based Au(I) metal complexes **96**–**100** as TrxR inhibitors with anticancer properties.

New NHC gold(I) complexes bearing a coumarin-type carbene ligand (**96**) and 1-thio-β-D-glucopyranosido groups as a second ligand (**97** and **98**) have been claimed as potential inhibitors of the TrxR enzyme by targeting the selenocysteine residue in the enzyme redox-active motif (Figure 42) [184]. Notably, complex **97** containing the tetra-*O*-acetyl-1-thio-β-D-glucopyranosido ligand was found to be more efficient in ovarian carcinoma (A2780) and breast carcinoma (MCF-7) cell lines (IC_50_ = 11.6–12.9 µM) than ligand **95** and complex **96** (IC_50_ = 39.7–71.2 µM). Moreover, in the case of MCF-7 cells, compound **97** exhibited better activity than *cisplatin* (IC_50_ = 12.9 µM vs. IC_50_ = 20.0 µM), whereas NHC gold(I) compound **96** bearing 1-thio-β-D-glucopyranosido turned out to be inactive in the in vitro cell viability assay. Further studies proved that compound **97** could inhibit cancer-relevant enzyme TrxR in A2780 cells by approximately 30% at a concentration of 10 µM (close to the IC_50_ value for an antiproliferative effect). Fluorescence microscopy analysis showed its ability to enter tumor cells and reach the nuclei, which may induce cell death [184].

In turn, alkynyl-gold(I) complexes **99** and **100** (Figure 42) with a propargyl-functionalized coumarin derivative exhibited moderate-to-strong inhibitory potency against TrxR activity (IC_50_ values ranging from 0.044 µM to >1 µM) [185]. Moreover, anionic complex **99** has been claimed to be the most effective in the treatment of HT-29 colon carcinoma and MDA-MB-231 breast cancer cell lines with IC_50_ values in a low micromolar range (IC_50_ = 2.13–4.08 µM) in comparison with ligands (IC_50_ >100 µM) and neutral complexes **100** (IC_50_ = 13.32–41.40 µM) [185].

## 7. Umbelliferone and 7-Hydroxycoumarin-Based Compounds as Probes and Sensors

Numerous synthetic coumarin derivatives may serve as fluorescent organic dyes due to their unique optical properties, i.e.*,* high quantum yields under physiological conditions, broad Stokes’ shifts, and photochemical stability. Coumarins possess the π-π conjugated system with electron-rich and charge transfer properties. This unique structure leads to their applications as a fluorophore. Due to their desirable photophysical properties, coumarins have been widely used in the development of specific sensors—pro-fluorophores, chemosensors, and chemodosimeters—which selectively detect various biological analytes [186].

Umbelliferone itself absorbs ultraviolet light strongly at several wavelengths, exerting fluorescence, and it may be applied as a fluorophore in designing optical devices. Moreover, different combinations of substitution patterns in the 7-hydroxycoumarin moiety do not destroy intense emissions; therefore, umbelliferone may serve as the core structure of these molecules [187].

Due to the fact that 7-hydroxycoumarin derivatives may exhibit multiple fluorescence controlled by the excitation wavelength, special attention has been paid to these types of fluorescent organic molecules as potent tools in designing bioprobes.

**Figure 43:**
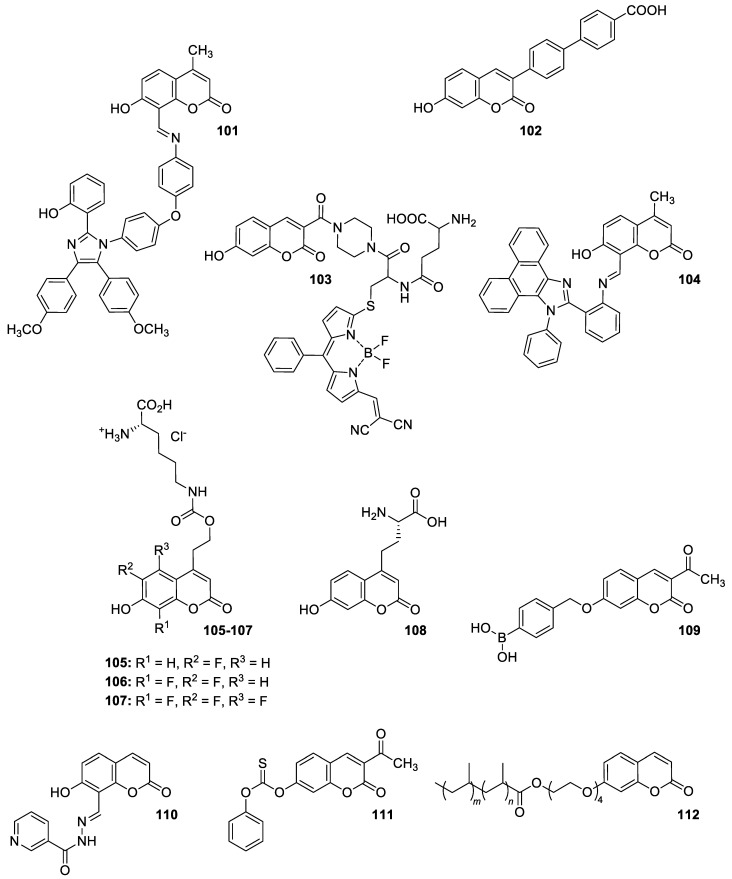
Chemical structures of 7-hydroxycoumarin-based compounds **101**–**112** with fluorescent properties.

Levin et al. [188] synthesized novel dyad molecule **101** shown in Figure 43 composed of two different fluorophores: 1,2,4,5-tetraarylimidazole and 8-arylazomethinocoumarin. Because of the presence of both proton (hydrogen) donating/accepting groups in the structure, the designed molecule exhibits multiple fluorescence with maxima at 450 nm and 535 nm as a result of excited-state intramolecular proton transfer (ESIPT). Compound **101** shows steady-state dual-fluorescence depending on the wavelength of photoexcitation below 400 nm in dichloromethane solution and polymethylmethacrylate film (PMMA) containing 0.05–0.25% of a luminescent compound. Interestingly, fluorescence intensity in a polymer is significantly higher than in a solution. It should be pointed out that systems based on ESIPT may have many applications, such as in numerous sensors, luminescent devices, or organic light-emitting diodes [188].

Recently, Xiao et al. focused on a series of 3-substituted umbelliferone derivatives in order to identify inhibitors of tautomerase of the macrophage migration inhibitory factor (MIF) with favorable physicochemical properties [189]. MIF is a pro-inflammatory cytokine which plays a pivotal role in the pathogenesis of many cancers. Its overexpression enhances angiogenesis, tumor growth, and progression. Hence, MIF enzymatic tautomerase activity has attracted considerable attention and displays a novel drug target for cancer treatment. The study demonstrated that selected 7-hydroxycoumarin derivative **102** (Figure 43) is a valuable tool in the advancement of MIF assays. Fluorogenic probe **102** displays clear and reversible fluorescence quenching upon binding to the MIF tautomerase active site (*K*_i_ = 18 nM). Compound **102** has favorable optical properties including a quantum yield of 0.32 and a Stokes shift of over 100 nm. Additionally, the relatively good solubility of probe **102** in PBS at pH 7.4 (18.8 μg/mL, 53 μM) results in regular assay data. It should be noted that available inhibitors have poor solubility under physiological conditions and high logP values, which may affect the assay readout. In the fluorescent indicator displacement (FID) assay, selected inhibitor **102** displayed good efficacy and potency in reducing fluorophore (50 nM) and MIF (100 nM) concentrations. Probe **102** was also found to disrupt the MIF-CD74 interaction and inhibit the growth of A549 cancer cells at a micromolar concentration [189].

In 2018, Shi et al. designed the switchable Förster resonance energy transfer (FRET) two-photon ratiometric probe (**103**) (Figure 43) for assaying γ-glutamyl transferase (GGT) activity, composed of 7-hydroxycoumarin that acts as an energy donor, a peptide derivative, and a 4,4-difluoro-4-bora-3*a*,4*a*-diaza-*s*-indacene (boron-dipyrromethene, BODIPY) moiety which is an energy acceptor [190]. Due to the short distance between the two fluorophores, in the free probe, FRET from the umbelliferone to BODIPY occurs efficiently. This gives two well-resolved emission bands at 461 nm and 610 nm, respectively. Upon interaction with the enzyme, the cleavage of the γ-glutamyl group and subsequent aromatic hydrocarbon transfer from the sulfur to nitrogen atoms leads to the rearrangement of the two fluorophores. This increases the distance between the two chromophores, contributing to a decrease in the FRET efficiency and the recovery of the donor fluorescence at 461 nm. The designed probe **103** may be applied to monitor γ-glutamyl transferase activity in living cells by two-photon fluorescence confocal microscopy utilizing GGT-triggered ratiometric responsiveness/measurement. It was claimed that the probe can differentiate ovarian cancer cells from normal cells by tracking GGT activity [190].

A new ‘off-on’ pH-sensitive fluorescent probe (**104**) for water solutions was synthesized by Li et al. starting from 2-(1-phenyl-1*H*-phenanthro [9,10-*d*]imidazol-2-yl)aniline and 7-hydroxy-4-methyl-2-oxo-2*H*-chromene-8-carbaldehyde (Figure 43) [191]. Schiff base **104** contains a donor (hydroxycoumarin) and π-acceptor (phenanthro [9,10-*d*]imidazole) conjugated system. It was observed that increasing the pH results in the enhancement of the intensity of fluorescence and it is not disturbed by the presence of common analytes. Moreover, the authors suggested that changes in absorption and emission spectra are the consequence of the various tautomeric forms of probe **104**. The enol form is present under neutral conditions, and it may be transformed in higher pH values to the form with the oxygen-centered anion and then to the stable keto form. The calculated hybrid **104** quantum yield in methanol by use of quinoline sulphate as a standard material is about *Φ* = 0.54 [191].

The site-selective incorporation of non-natural fluorescent amino acids into proteins has proven useful for studying cell processes. Designed probes need to work efficiently at all cell locations. Compounds with enhanced acidity and desirable optical properties in acidic environments can be used to assess biological systems that function under reduced pH conditions, for example, lysosomes or endosomes.

Shukla’s research group described the scalable synthesis of fluorinated 7-hydroxycoumarin-functionalized lysines which may find use in probing protein function in acidic environments [192]. Fluorinated lysine derivatives **105**–**107** shown in Figure 43 when excited at 360 nm exhibit fluorescence (*Φ*_f_ = 0.58–0.70) at pH values lower than 6 (p*K*_a_ = 4.0–6.2).

In 2020, Gleason et al. demonstrated for the first time that fluorescent non-canonical amino acid (fNCAA) containing a 7-hydroxycoumarin moiety (**108**, Figure 43) can be used as an acceptor of Förster resonance energy transfer (FRET) in a single protein containing multiple tryptophan residues [193]. The fNCAA based on L-(7-hydroxycoumarin-4-yl)ethylglycine (**108**) due to its small size may be easily incorporated into versatile sites of proteins [194]. To examine the utility of the tryptophan/L-(7-hydroxycoumarin-4-yl)ethylglycine (**108**) pair, the dependence of enzyme activity—hexokinase—on pH in the presence of a substrate was assessed. As the glucose concentrations increased from 100 nM to 10 mM, an increase in the fluorescence intensity at 450 nm was observed. The obtained results suggest the utility of this system for studying protein function coupled to conformational changes. Such FRET pair systems may find uses in high-throughput screening or monitoring drug metabolites expanding the versatility of FRET-based techniques [194].

Ratiometric fluorescent biosensors are also constructed utilizing metal nanoclusters due to their low toxicity and enhanced photostability. Some of these platforms contain thiol-stabilized copper aggregates [195,196]. Due to their unique properties of aggregation-induced emission (AIE), their lifespans are prolonged [197].

A novel approach is the addition of metal cations which may enhance the aggregation-induced emission phenomenon. Fluorescence probe sensitive to hydrogen peroxide and glucose utilizing copper nanoclusters accompanied by cerium and iron ions (CuNCs-Ce^3+^/Fe^2+^) was designed by Mei et al. [198]. This assay is based on a known photocatalytic reaction where in the presence of hydrogen peroxide and generated ‘in situ’ hydroxyl radicals, a non-fluorescent coumarin is transformed into a fluorescent derivative—umbelliferone [199,200]. In the presence of H_2_O_2_, the red fluorescence of copper nanoclusters at 625 nm was quenched. In contrast, blue fluorescence at 460 nm dramatically increased due to the hydroxylation reaction of coumarin. The designed fluorescent assay is quick, eco-friendly, inexpensive, and simple to operate, changing its color.

Recently, it was reported that sensors based on the oxidation of organic borates have superior selectivity for the detection of hydrogen peroxide than other ROS. In 2023, novel probe **109** (Figure 43) composed of 3-acetyl-7-hydroxy-2*H*-chromen-2-one and arylboronic acid for monitoring hydrogen peroxide levels in biological systems was synthesized in two steps by Wang et al. [201]. Upon the reaction of hydrogen peroxide with the arylboronic acid moiety, compound **109** transforms into a derivative containing an electron-donating hydroxyl group, which in the presence of the 3-acetyl electron-sucking group forms a push–pull system. The product of this reaction—3-acetyl-7-hydroxycoumarin—displays a strong fluorescence emission and may act as a specific recognition group in hydrogen peroxide detection. Probe **109** was shown to be selective towards hydrogen peroxide because only the reaction of hydrogen peroxide produced significant fluorescence under phosphate buffered saline (PBS). Therefore, this probe provides a potential and highly selective biomedical tool for monitoring various diseases caused by an excess of hydrogen peroxide [201].

In 2018, Zhu et al. reported the synthesis of novel 7-hydroxycoumarin chemosensor **110** (Figure 43) in a straightforward manner by refluxing 8-formyl-7-hydroxycoumarin with nicotinohydrazide in ethanol [202]. Compound **110** characterized a greater fluorescence enhancement toward aluminum ions in ethanol-HEPES buffer solution (pH = 7.4) due to the photoinduced electron transfer (PET). The fluorescence intensity of the **110**-Al^3+^ complex was measured at 463 nm. The study shows that fluorescent chemosensor **110** may serve as a highly selective tool for Al^3+^ detection in biological systems with no disturbance from other metal ions.

Recently, Li et al. have reported 7-hydroxycoumarin-based carbonothioate derivative **111** (Figure 43) as a highly sensitive fluorescent probe for mercury ions with many practical applications [203]. The carbonothioate moiety acts as a selective and specific binder for Hg^2+^, and due to the good water solubility of probe **111,** it can be used in cells or biological samples. The detection limit of 7.9 nM for probe **111** was determined from the fluorescence titration. The detection of mercury is quantitative over the concentration range of 0–2 µM.

Rojas-Montoya et al. designed a series of novel grafted photoluminescent polymers (**112**) (Figure 43) by gamma irradiation of polyethylene in the presence of acryloyl chloride, followed by an esterification reaction with a 7-hydroxycoumarin derivative functionalized with flexible chains of tetraethylene glycol [204]. Coumarin-tetra(ethylene glycol) derivatives were incorporated from 1.2% to 15.8% in a matrix of polyethylene (PE). In order to obtain the emission spectra of the grafted PE in solid films, they were excited at 323 nm and the emission spectra were measured. Increasing the radiation dose to the sample resulted in a significant increase in fluorescence intensity. The obtained photoluminescent polymers (**112**) show a maximum absorption wavelength of 323 nm and emission wavelength of 394 nm. This methodology may provide a wide variety of photoluminescent polymers useful as blue-emitting luminescent devices [204].

## 8. Conclusions

Umbelliferone (UMB, 7-hydroxycoumarin) is a natural coumarin-derived compound with a diversity of bioactivities, including anti-inflammatory and antioxidant properties, disease prevention, cell growth modulation, and enzyme inhibition, among others. A large number of research groups have revealed that UMB possesses a promising pharmacological and safety profile, and it could be expected to treat various diseases such as inflammation, neurodegenerative disorders, neuropsychiatric diseases, diabetes, cancer, and microbial infections [3,4,5]. Additionally, the efficiency of the synthetic routes to obtain a wide range of functionalized 7-hydroxycoumarins with a variety of activities ensures that umbelliferone is an inspiring scaffold in drug design and development [3,6,7,8,205,206].

The objective of this review was to provide a perspective on the discovery of novel 7-hydroxycoumarin-derived compounds including their metal complexes with potential for therapeutic applications as anti-inflammatory, antioxidant, antineurodegenerative, antipsychotic, antiepileptic, antidiabetic, and chemotherapeutic agents. In this review, we presented the results from the investigations reported in the literature mainly in the period of 2017–2023.

Umbelliferone demonstrated beneficial anti-inflammatory and antioxidant properties through various mechanisms. Considering the importance of UMB, researchers extensively explored it by synthesizing novel 7-hydroxycoumarin-based compounds as anti-inflammatory and antioxidant agents. In addition to structure–activity analysis, their effects on various inflammatory targets such as IL-6 and TNF-α by blocking the MAPK- and/or NK-κB signaling pathways were highlighted.

Another valuable feature of umbelliferone is that it may constitute a starting point for the development of agents acting in the CNS. Several series of 7-hydroxycoumarin-derived compounds have been reported as potent selective enzyme inhibitors and as dual-acting agents, as well as multifunctional ligands, towards neurodegenerative or neuropsychiatric disorders. The influence of the type of substituent and substitution pattern on the biological effects of the investigated compounds was analyzed, indicating the most pronounced molecules.

Other diseases where the therapeutic potential of parent 7-hydroxycoumarin and its derivatives may have a significant contribution are microbial infections and cancer.

In terms of antimicrobials, it is noteworthy that UMB exerted significant selectivity for inhibiting the bacterial over the human isoforms hCA I and II, which may be useful for the development of antibacterial agents towards a novel therapeutic target that can revert the drug resistance observed with the clinically used antibiotics. Moreover, SAR studies suggest that the substitution at C-3, C-7, or C-8 of the parent molecule is most important for the antimicrobial effects.

There are many examples of 7-hydroxycoumarin-based compounds targeting specific enzymes, receptors, and proteins involved in the pathogenesis of various types of human tumors, leading to apoptosis and inhibition of cancer cell proliferation. Considerable progress in this field has been achieved through the rational design of molecules containing a 7-hydroxycoumarin nucleus. In this regard, numerous 7-hydroxycoumarin derivatives have been reported as histone deacetylase inhibitors, androgen receptor antagonists, inhibitors of the PIK3/Akt signaling pathway, tyrosyl-DNA phosphodiesterase 1 or carbonic anhydrase inhibitors, and cyclooxygenase-2 and 5-lipoxygenase inhibitors.

7-hydroxycoumarin-based metal complexes have also attracted attention because coordination offers opportunities for the development of more potent molecules. Therefore, the recent progress in the development of 7-hydroxycoumarin-based metal complexes as antioxidant, antimicrobial, and anticancer agents has been summarized. In this context, 7-hydroxycoumarin-platin and –ruthenium complexes are of particular interest as a promising class of chemotherapeutics which may demonstrate a specific mode of action in cancer therapy.

Furthermore, in addition to potential therapeutic applications, the fluorescence properties of 7-hydroxycoumarin-based compounds and their practical applications as sensors and probes in biological species have also been discussed.

In conclusion, the presented studies highlight the significant role of umbelliferone and umbelliferone-derived compounds in drug design and development, although they need further research. Nevertheless, there is no doubt that 7-hydroxycoumarins constitute an important source of potential new therapeutics. The selected compounds included in the review are presented in Table 1.

## Figures and Tables

**Table 1:** Examples of 7-hydroxycoumarin-based compounds included in the review, summarizing the biological activity and molecular target.

Structure	Biological Activity	Molecular Target	Name/ Number	Ref.
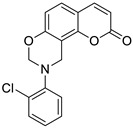	Anti-inflammatory	MAPK and NK-κB	1a	[32]
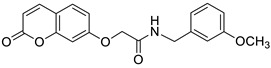	Anti-inflammatory	NK-κB p65	2a	[34]
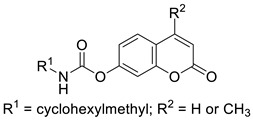	Antioxidant and anti-Alzheimer’s disease (anti-AD)	Free radicals and BuChE	7	[41]
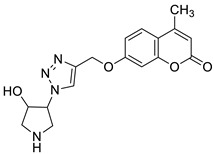	Antioxidant	Free radicals	8a	[42]
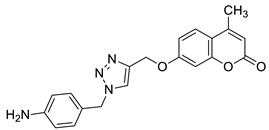	Antioxidant	Free radicals	8b	[42]
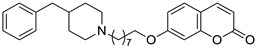	Antioxidant	Free radicals	10	[44]
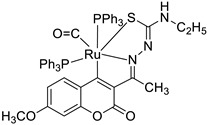	Antioxidant	Free radicals	11	[47]
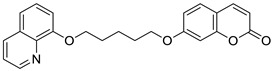	Anti-Alzheimer’s disease (anti-AD)	AChE and BuChE	17	[60]
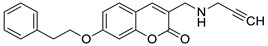	Anti-Alzheimer’s disease (anti-AD) and neuroprotective	MAO-B	18	[63]
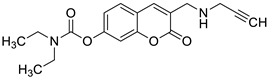	Anti-Alzheimer’s disease (anti-AD) and neuroprotective	MAO-B	19	[63]
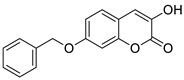	Treatment of neuropsychiatric diseases (schizophrenia)	DAAO	22	[69]
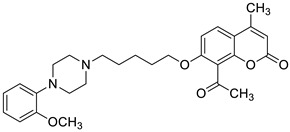	Treatment of neuropsychiatric diseases	5-HT_1A_	23	[70]
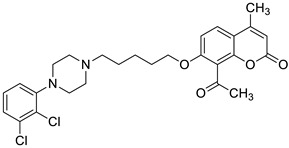	Treatment of neuropsychiatric diseases	5-HT_2A_	24	[70]
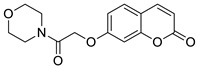	Antiepileptic	GABA_A_	25	[73]
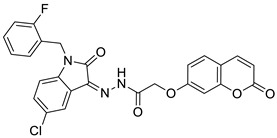	Antidiabetic	α-glucosidase	26	[79]
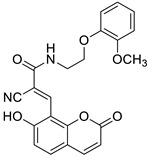	Antibacterial and antifungal	*E. coli*, *S. aureus*, *P. aeruginosa*, *A. niger*, and *C. albicans*	27	[88]
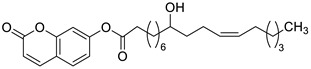	Antibacterial and antifungal	*B. subtilis*, *S. pyogenes*, *S. aureus*, *E. coli*, *C. albicans*, *C. parapsilosis*, and *C. neoformans*	31	[91]
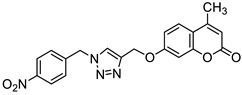	Antibacterial	*M. luteus*, *B. cereus*, *E. coli*, and *P. fluorescens*	34a	[95]
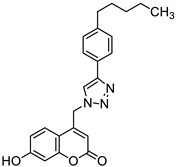	Antibacterial	VRA *E. faecium* and *E. faecalis*	35	[96]
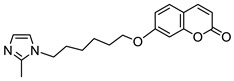	Antibacterial and antiviral	*E. coli* and infectious hematopoietic necrosis virus (IHNV)	41	[97] [111]
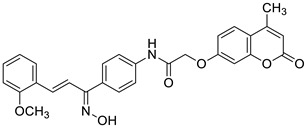	Antibacterial	*S. aureus*	45	[98]
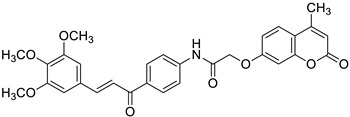	Antibacterial	*S. aureus*, *E. coli*, and *K. pneumoniae*	46	[98]
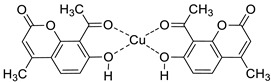	Antibacterial and antifungal	*S. aureus*, *B. subtilis*, *B. cereus*, *S. epidermis*, *P. aeruginosa*, and *C. albicans*	50	[102]
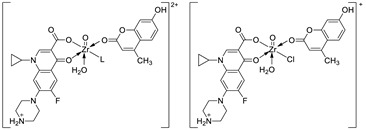	Antibacterial	*B. subtilis*, *B. cereus*, *P. aeruginosa*, and *E. coli*	CIP—HMC	[103]
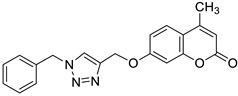	Antitubercular	*M. tuberculosis* H37Ra and Dpr E1	51	[104]
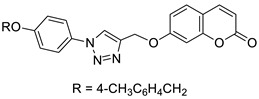	Antimalarial	*P. falciparum*	53	[43]
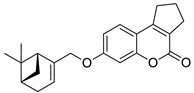	Antiviral	Influenza A virus (viral hemagglutinin, proton channel M2)	54	[110]
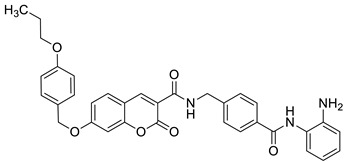	Antiproliferative	Colon cancer cell line (HCT116), lung cancer cell line (A549), and leukemia (HL60)	55	[131]
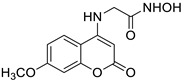	Anticancer	HDAC1	59	[132]
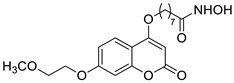	Anticancer	HDAC1	61	[133]
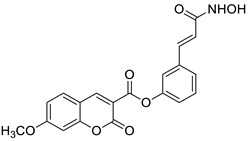	Anticancer	HDAC1 and cervical cancer cell line (HeLa)	65	[134]
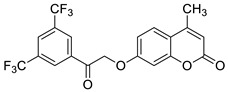	Anticancer	Prostate cancer cell line (22Rv1) and breast cancer cell line (MCF-7)	68	[138]
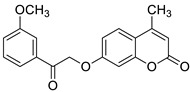	Anticancer	Prostate cancer cell line (22Rv1) and breast cancer cell line (MCF-7)	69	[138]
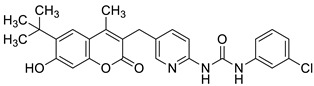	Anticancer	PI3Kα/β/δ signal pathway, lung carcinoma (A549), breast carcinoma (MCF-7), leukemia (K562), and cervical carcinoma (HeLa)	72	[144]
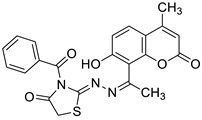	Cytotoxic	PI3Kα/Akt-1 signal pathway and breast carcinoma (MCF-7)	75	[145]
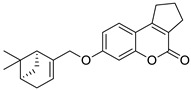	Anticancer	Tyrosyl-DNA phosphodiesterase (Tdp1)	77	[152]
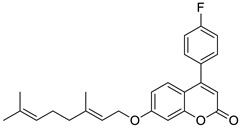	Anticancer	Krebs-2 carcinoma	78	[153]
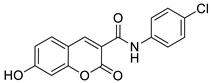	Anticancer	CA IX and CA XII	80	[162]
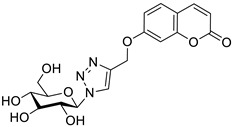	Anticancer	CA IX	82a	[167]
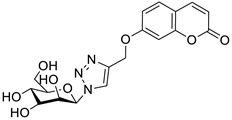	Anticancer	CA IX	82b	[167]
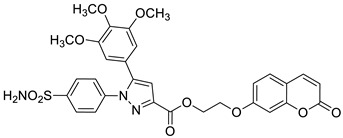	Anticancer	COX-2, 5-LOX, and lung carcinoma (A549)	83	[174]
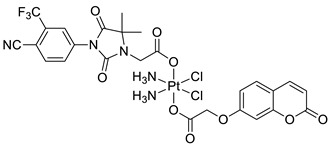	Anticancer	Androgen receptor (AR) and prostate adenocarcinoma	85	[177]
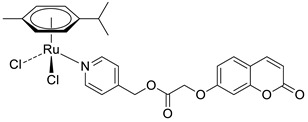	Anticancer	ERK signal pathway, colorectal cancer (HCT-116), HepG-2 (hepatocellular carcinoma), and non-small cell lung cancer (A549)	92	[182]
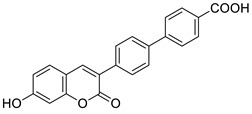	Fluorescent sensor	MIF tautomerase active site	102	[189]
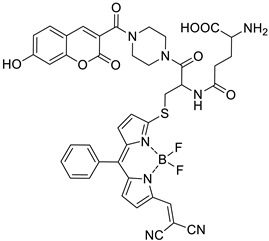	Two-photon ratiometric probe	γ-glutamyl transferase (GGT)	103	[190]
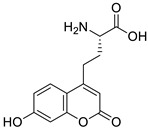	Fluorescent non-canonical amino acid (fNCAA)	Acceptor of FRET in HTS or monitoring of drug metabolites	108	[194]
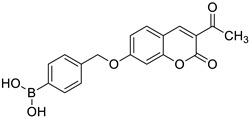	Fluorescent probe	H_2_O_2_	109	[201]
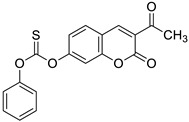	Fluorescent probe	Hg^2+^	111	[203]

## Data Availability

Data sharing is not applicable.
